# A family of archaea-like carboxylesterases preferentially expressed in the symbiotic phase of the mycorrhizal fungus *Tuber melanosporum*

**DOI:** 10.1038/s41598-017-08007-9

**Published:** 2017-08-09

**Authors:** Davide Cavazzini, Guido Grossi, Elisabetta Levati, Francesca Vallese, Barbara Montanini, Angelo Bolchi, Giuseppe Zanotti, Simone Ottonello

**Affiliations:** 1 Department of Chemical Life Sciences and Environmental Sustainability, University of Parma, Parco Area delle Scienze 23/A, 43124 Parma, Italy; 2 Department of Biomedical Sciences, University of Padova, Via Ugo Bassi 58/B, Padova, 35131 Italy; 3 Present Address: Interdisciplinary Nanoscience Center (iNANO), Aarhus University, Gustav Wieds Vej 14, 8000 Aarhus, Denmark

## Abstract

An increasing number of esterases is being revealed by (meta) genomic sequencing projects, but few of them are functionally/structurally characterized, especially enzymes of fungal origin. Starting from a three-member gene family of secreted putative “lipases/esterases” preferentially expressed in the symbiotic phase of the mycorrhizal fungus *Tuber melanosporum* (“black truffle”), we show here that these enzymes (TmelEST1-3) are dimeric, heat-resistant carboxylesterases capable of hydrolyzing various short/medium chain p-nitrophenyl esters. TmelEST2 was the most active (*kcat* = 2302 s^−1^ for p-nitrophenyl-butyrate) and thermally stable (T_50_ = 68.3 °C), while TmelEST3 was the only one displaying some activity on tertiary alcohol esters. X-ray diffraction analysis of TmelEST2 revealed a classical α/β hydrolase-fold structure, with a network of dimer-stabilizing intermolecular interactions typical of archaea esterases. The predicted structures of TmelEST1 and 3 are overall quite similar to that of TmelEST2 but with some important differences. Most notably, the much smaller volume of the substrate-binding pocket and the more acidic electrostatic surface profile of TmelEST1. This was also the only TmelEST capable of hydrolyzing feruloyl-esters, suggestinng a possible role in root cell-wall deconstruction during symbiosis establishment. In addition to their potential biotechnological interest, TmelESTs raise important questions regarding the evolutionary recruitment of archaea-like enzymes into mesophilic subterranean fungi such as truffles.

## Introduction

Lipolytic enzymes, including esterases (EC 3.1.1.1) and lipases (EC 3.1.1.3), catalyze the cleavage and formation of ester bonds^1,2^. Because of their activity on a broad range of non-natural substrates, high enantioselectivity, lack of cofactor requirements and a generally high organic solvent and thermal stability, these enzymes are very attractive industrial biocatalysts, including “green chemistry” applications such as bio-based polyester synthesis and biodiesel production^3–6^.

Lipases act on water-insoluble, long-chain fatty acid esters, display interfacial activation and are functionally distinct from carboxylesterases, which are instead specialized in the hydrolysis of short/medium (C < 10) chain carboxylic acid esters^4,7^. Based on amino acid sequence similarity and conservation of particular sequence motifs, lipolytic enzymes are classified into four major blocks (C, H, L and X)^8^. The Hormone Sensitive Lipases (HSL) family belongs to block H and comprises multiple esterases and lipases from very diverse organisms and with extremely varied substrate preferences^8^. Originally identified in mammals, HSL enzymes have subsequently been found also in a variety of microbes, including fungi, where they mainly act as esterases, rather than lipases. Specifically, bacterial enzymes belonging to family IV, one of the eight families of bacterial lipolytic enzymes originally defined on the basis of sequence conservation and biological properties criteria^2^, closely resemble HSL enzymes^9^. These are further subdivided into two subfamilies, the most common of which contain characteristic GDSXG or GTSXG sequence motifs^10,11^. All HSL esterases (EST) identified so far, display a characteristic α/β hydrolase fold^12^, with a central β-sheet surrounded by multiple α-helices. This serves as a stable core bearing most of the amino acid substitutions, loop insertions and deletions that have contributed to catalytic function diversification during evolution. The active site is generally composed of a catalytic triad consisting of Ser, Asp/Glu and His residues, with the Ser nucleophile usually inserted into the conserved GDSXG consensus motif^9^ within the so called “nucleophile elbow”^12^. Another conserved motif is the His-Gly-Gly-Gly sequence, which is located approximately 70 amino acids upstream from the catalytic serine residue and forms the “oxyanion-hole”^7^. Several HSL-ESTs from various microorganisms and metagenomic libraries have been described in recent years^13,14^. The three-dimensional (3D) structures of a few of them have also been determined, with a marked prevalence of bacterial enzymes^15–19^ compared to fungal HSL-ESTs^20^, as it is the case for other protein families. All microbial HSL-ESTs structurally characterized so far share the α/β-fold signature, have molecular masses comprised between 34 and 40 kDa and many of them (especially esterases from extremophiles) are present in solution as dimers or other higher-order oligomeric forms.

Three distinct genes automatically annotated as “secreted lipases/esterases”^21^ are preferentially expressed in the symbiotic phase of *Tuber melanosporum* (also known as “black truffle”), a mycorrhizal ascomycete whose genome and methylome have been sequenced^21,22^. This fungus, which forms mutualistic interactions with various host-plants, is characterized by a strictly subterranean habitat and three distinct lifecycle stages: free-living mycelium (FLM), fruiting bodies (FB; also known as “truffles”) and the ectomycorrhizal (ECM) symbiotic stage.

We present here the results of a detailed functional characterization and 3D structure analysis of these three *T*. *melanosporum* enzymes, designated as TmelEST 1, 2 and 3, which are shown to be non-lypolitic HSL carboxylesterases closely resembling archaeal and endosymbiotic soil bacteria esterases. Structural analysis combined with site-directed mutagenesis experiments, also allowed to pinpoint some novel features of the structure-function relationships of microbial HSL esterases.

## Results

As revealed by microarray analysis^21^ and confirmed by RNAseq transcriptome profiling^23^, the three TmelEST genes are preferentially expressed in ectomycorrhizae, with the highest relative expression levels for TmelEST3, followed by TmelEST1 and TmelEST2 (see the outline of expression data in Table S1). The latter gene is expressed at 50% lower levels in ECM compared to TmelEST3, but is the most expressed in both free-living mycelium and fruitbodies. A fourth putative TmelEST gene (GSTUMT00012317001, scaffold_13:437727-438838) was identified at the beginning of this work, but due to its barely detectable expression levels and location in a highly methylated, transposon-rich region of the *T*. *melanosporum* genome^22^, it was classified as a pseudogene and excluded from further analysis.

The predicted TmelEST polypeptides are on average 81% similar to each other, with maximal similarity between TmelEST1 and 2. The secretory nature of the TmelEST proteins, which contain a predicted secretion signal peptide encompassing the first 18 N-terminal amino acids, was confirmed by SignalP-4^24^ and TargetP 1.1^25^ (secretion probability scores for the three proteins ranging from 94.5% to 96.6%).

**Figure 1:**
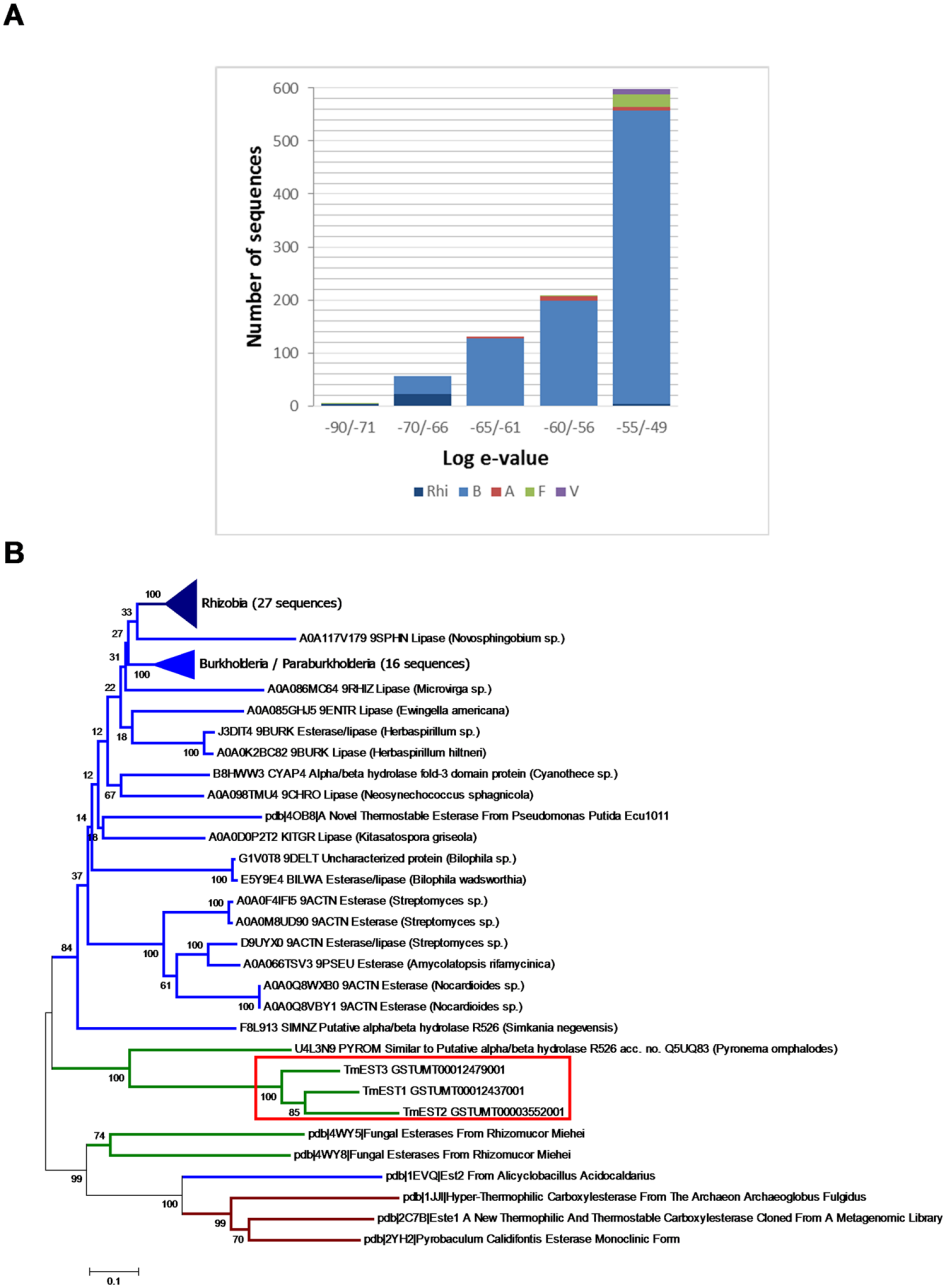
TmelEST homologs and phylogeny. (**A**) The top 1,000 best hits of the TmeEST proteins, retrieved from a BLAST-P search at the UniProt database (release 2016_08), are classified according to their e-value ranges (from e^−90^ to >e^−49^) as indicated. Bacterial hits are in *blue* (*dark blue* for Rhizobia), Archaea are in *red*, fungal hits in *green* and viral hits in *purple*. (**B**) Neighbor-joining phylogenetic analysis based on the alignment of the highest-scoring hits retrieved from the UniProt (e-value ≤ 10^−66^, a total of 61 sequences; see Fig. S1 for an alignment restricted to the TmelESTs and their five best-homologs) and the PDB databases (7 sequences); TmelEST sequences are red-boxed. Bootstrap values are shown next to the branches; the scale bar indicates evolutionary distances.

Nearly all TmelEST best homologs are of bacterial origin, with a prevalence of hyperthermophilic archaea and endosymbiotic Rhizobia, and only one fungal homolog from the saprotroph *Pyronema omphalodes*. As shown in Fig. 1A, 96% of the TmelEST homologs revealed by a BLAST-P search conducted in the UniProt database and limited to the first 1,000 top hits are of Prokaryotic origin (944 Bacteria and 18 Archaea).

A few structural homologs of the TmelESTs (with sequence identity values ranging from 31 to 42% and e-values ranging from 10^−60^ to 10^−30^; see Table S2) were also retrieved from a search in the PDB database. In accordance with the BLAST-P results, the best hits revealed by this structure-based analysis are thermostable esterases belonging to the HSL superfamily, again with a large prevalence of bacterial enzymes. The only fungal structures are two carboxylesterases (RmEstA and B) from the thermophilic zygomycete *Rhizomucor miehei*^20^, one of which (RmEstB) was among the three top hits, preceded by the thermophilic esterase rPPE from *Pseudomonas putida*^18^. Two other structural homologs found among the five top hits, are the thermophilic esterases PestE and AFEst from the archaea *Pyrobaculum calidifontis*^17^ and *Archaeoglobus fulgidus*^26^.

As revealed by the phylogenetic tree in Fig. 1B, which was derived from the alignment of the highest-scoring hits (e-values comprised between 10^−90^ and 10^−66^) retrieved from the UniProt and the PDB databases, TmelESTs form a separate cluster close to a large branch exclusively occupied by bacteria.

As shown in Fig. S1, even though the TmelEST homologs revealed by structural similarity analyses are not the best-hits in absolute terms, they can all be aligned with the *T*. *melanosporum* proteins, including the consensus sequence Gly-Asp-Ser-X-Gly, the other two active site residues in addition to Ser (His and Asp) and the oxyanion-hole sequence motif His-Gly-Gly-Gly. The most conserved is the central region containing the HSL sequence pattern, whereas the N-terminal region, which is known to affect substrate specificity^27^, appears to be the most divergent.

### TmelESTs are dimeric esterases preferentially acting on short-chain carboxylic acid esters

**Figure 2:**
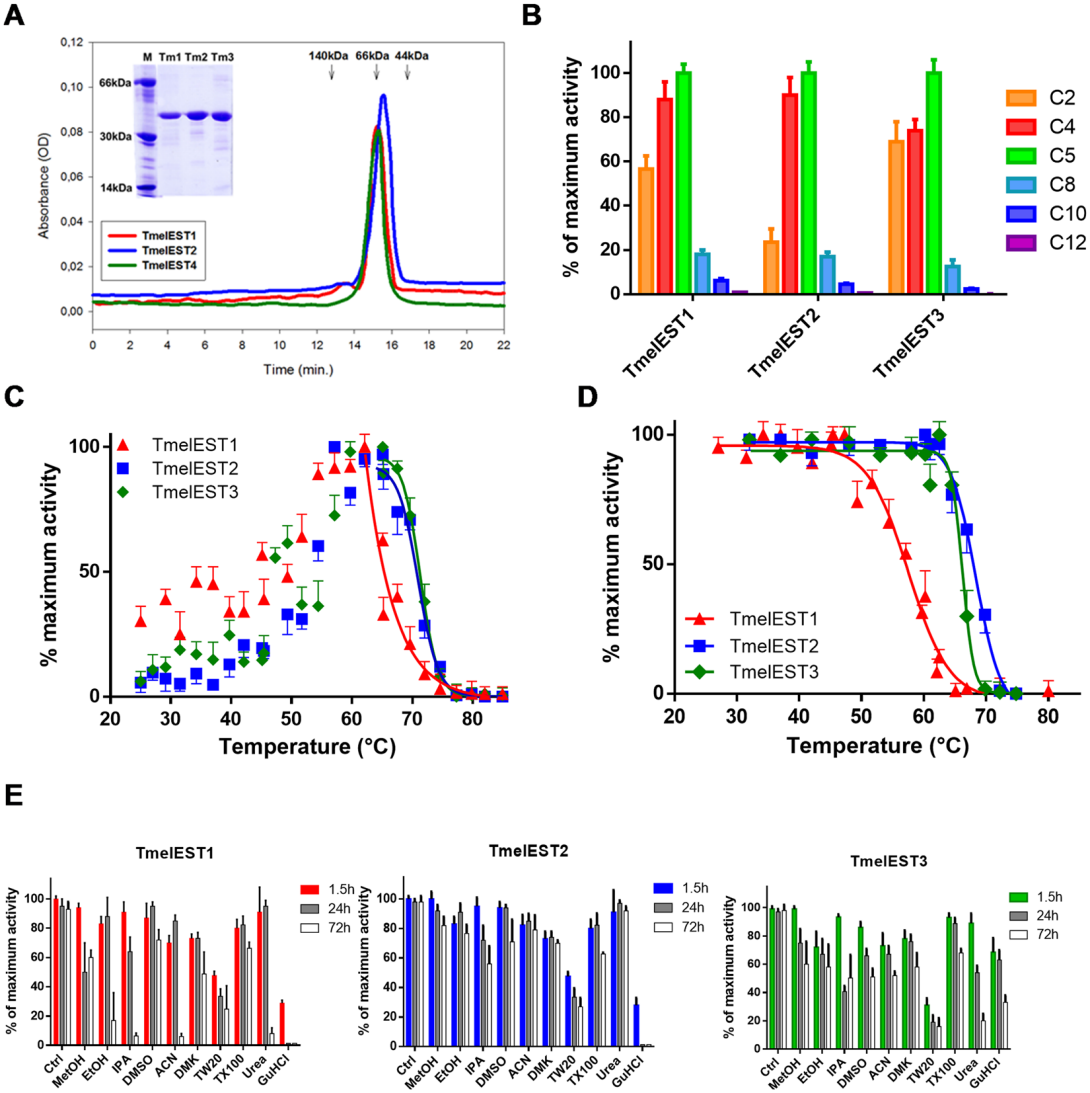
Biochemical characterization of recombinant TmelESTs. (**A**) Dimeric native state and purity of the TmelEST proteins assessed by gel filtration analysis (Superdex 200 column); the elution times of molecular mass standards are indicated. The subunit molecular weight and purity of the TmelEST as revealed by SDS-PAGE are show in the inset. (**B**) Substrate preference of TmelESTs for pNP-esters with different acyl-chain lengths. Enzyme activity, measured under standard reaction conditions (see ‘Methods’ for details) in the presence of the indicated pNP-esters, is expressed as percent of maximum activity; p-nitrophenyl palmitate (C16), whose hydrolysis was practically not detectable, was omitted. (**C**) Optimum reaction temperature of the TmelESTs determined by enzyme activity assays conducted at the indicated temperatures (25–80 °C) under standard reaction conditions (**D**) Thermal stability of the TmelESTs measured by incubation for 5 min at increasing temperatures, followed by enzyme activity determination at 25 °C under standard reaction conditions. Under identical experimental conditions, palatase and CalB, two commercial lipases utilized for various bio-industrial applications yielded T_50_ values of 51.6 and 54.3 °C, respectively (**E**) Effect of the indicated organic solvents, detergents and denaturants on TmelEST activity. Residual activity of each enzyme was measured under standard reaction conditions after incubation for the indicated times (1.5, 24 and 72 h) in the presence of a 30% (v/v), 5% (v/v) and 2.5 M concentrations of the indicated organic solvents (MetOH: methanol; EhOH: ethanol; IPA: isopropyl alcohol; DMSO: dimethylsulfoxide; ACN: acetonitrile; DMK: acetone), detergents (TW20: Tween 20; TX100: Triton X-100) and denaturants (Urea, GuHCl: guanidium hydrochloride).

Following isolation of the corresponding cDNAs from *T*. *melanosporum* ECM and FLM libraries, individual TmelESTs were expressed in *E*. *coli* and purified by metal-affinity chromatography. As revealed by SDS-PAGE (carried out after His-tag removal; Fig. 2A inset) and MALDI-TOF mass spectrometry analysis (not shown), recombinant Tmel-ESTs, from which the signal peptide sequence has been removed, have a subunit molecular mass (~35 kDa) typical of carboxylesterases. A nearly double molecular size (~65 kDa), which did not change upon β-mercaptoethanol supplementation, was derived from gel-filtration analyses carried out under non-denaturing conditions (Fig. 2A). In the native state, TmelESTs are thus present in a dimeric, non-disulfide-bonded form as observed for other HSL esterases, most notably the similarly sized hyperthermophilic EstE1 enzyme derived from a metagenomic library^28^.

A series of *p*-nitrophenyl (*p*NP)-carboxylic acid esters (C2–C16) was used to assess the catalytic competence and substrate preference of the TmelESTs. As shown in Fig. 2B, specific activity increased with increasing acyl-chain length up to C5 (*p*NP-valerate; *p*NPV), followed by a sharp decrease with longer acyl-chain substrates. *p*NPV was converted at almost the same rate as *p*-NP-butyrate (*p*NPB) by TmelEST2, while *p*-NP-acetate (*p*NPA) was hydrolyzed as fast as *p*NPB by TmelEST3. The lack of activity on longer acyl-chain *p*NP-esters, the absence of interfacial activation (i.e., no activity on micellar *p*NPB; data not shown) and the lack of activity on medium chain triacylglycerols (tricaproin, glyceryl trioctanoate and tricaprin; not shown), all support the notion that TmelESTs are non-lypolitic carboxylesterases^4^.

The GGG(A)X oxyanion-hole motif has been proposed to be a key determinant of hydrolytic activity on tertiary alcohol esters (TAE)^29,30^, which are the building blocks for various organic syntheses. TmelEST2 and 3 bear an HGGGW motif, which slightly differs from the HGGAW motif present in TmelEST1. Using a modified assay procedure based on the measurement of hydrolysis-dependent proton release^31^, we tested TAE hydrolytic activity on linalyl acetate, tert-butyl acetate and α-terpinyl acetate. Only a modest activity on linalyl acetate, the least bulky of the three TAE substrates, was observed with TmelEST3 (0.1 µmol/min/mg protein; 90 min incubation time), whereas hydrolysis of α-terpinyl acetate by the same enzyme required a much longer incubation time.

All TmelESTs exhibited a pH optimum around 7.0 and followed Michelis-Menten kinetics (data not shown). A linear time-dependence was observed with short-chain esters (*p*NPA and *p*NPB), whereas hydrolysis of longer acyl-chain esters (C8–C10) displayed a biphasic kinetics (Fig. S2A,B). As shown in Table 1, the K_m_ values for *p*NPB and *p*NPV ranged from 45 to 160 µM and with both substrates they were 2–3-fold lower for TmelEST2 compared to the other two esterases. More striking was the difference in *kcat*, which was 30-fold and 100-fold higher for TmelEST2 compared to TmelEST3 and 1, respectively. In fact, the *kcat* for *p*NPB hydrolysis of TmelEST2 (2302 s^−1^) and even more so the *kcat*/K_m_ ratio (46982 mM^−1^ s^−1^) are among the highest thus far described for HSL esterases, including the highly efficient PestE and Est22 enzymes^17,32^. A similar ranking of hydrolytic activity was determined on 1-naphtyl acetate, with apparent V_max_ values of 40, 15 and 0.03 µmol/min/mg of protein for TmelEST 2, 3 and 1, respectively.

**Table 1:** Kinetic parameters for pNP-butyrate (C4) and pNP-valerate (C5) hydrolysis by the TmelEST carboxylesterases.

Enzyme	Substrate	Vmax µmol/min/mg	K_m_ mM	*kcat* s^−1^	*kcat*/K_m_ mM^−1^ s^−1^
TmelEST1	C4	35 ± 2	0.140 ± 0.014	21.1	150.7
	C5	48.3 ± 2	0.160 ± 0.020	29.1	181.8
TmelEST2	C4	3840 ± 180	0.049 ± 0.008	2302.1	46982
	C5	4794 ± 183	0.044 ± 0.007	2874.1	65320
TmelEST3	C4	135 ± 2.5	0.109 ± 0.006	82.2	754.1
	C5	238 ± 11	0.137 ± 0.020	144.9	1057.7

### Thermal and organic solvent stability of the TmelESTs

As predicted by their similarity to esterases from extremophiles, TmelESTs proved to be heat-resistant, with temperature optima for *p*NPB hydrolysis ranging from 71 °C (TmelEST2 and 3) to 66 °C (TmelEST1) (Fig. 2C). TmelEST2 and 3 remained fully active after a 72 h incubation at 50 °C, whereas a complete loss of activity was observed for TmelEST1 after 24 h at the same temperature (not shown). As revealed by an additional experimental set-up, based on a 5 min incubation at increasing temperatures (25–85 °C) followed by *p*NPB hydrolysis assays, TmelEST2, with an apparent T_50_ of 68.3 °C, is the most thermally stable of the three *Tuber* esterases, followed by TmelEST1 (66.2 °C) and TmelEST3 (58.7 °C) (Fig. 2D).

As further shown in Fig. 2E, TmelEST2 was also the most resistant to a variety of organic solvents and detergents, including various alcohols, which at a fixed 30% (v/v) concentration only caused a 20% decrease of enzyme activity after a 72 h incubation, compared to a 50% and an 80% loss of activity for TmelEST3 and 1, respectively. As expected for hydrolytic enzymes bearing a catalytic serine residue, TmelESTs were all irreversibly inhibited by phenylmethylsulfonyl fluoride (PMSF, 2 mM; data not shown).

In view of biotechnological applications requiring large amounts of a high-performance, easy to purify and endotoxin-free enzyme, we also set-up a procedure for the secretory production of TmelEST2 in the metylotrophic yeast *Pichia pastoris*^33^ (see Supplementary Methods).

### Feruloyl-esters as potential TmelEST1 substrates

A small set of glycoside hydrolases (GH), including cellulases and pectinases that are thought to be instrumental to plant cell wall deconstruction during root apoplast invasion and colonization, is encoded by the *T*. *melanosporum* genome^21,34^. Hydroxycinnamoyl esters formed by phenolic acids (most notably, ferulic acid) cross-link different cell wall components (hemicellulose and pectin, but also lignin) thus greatly stabilizing cell wall architecture. Hydrolytic cleavage of these cross-links by secreted feruloyl esterases is required to loosen plant cell walls and give access to cellulolytic and pectinolytic enzymes.

**Figure 3:**
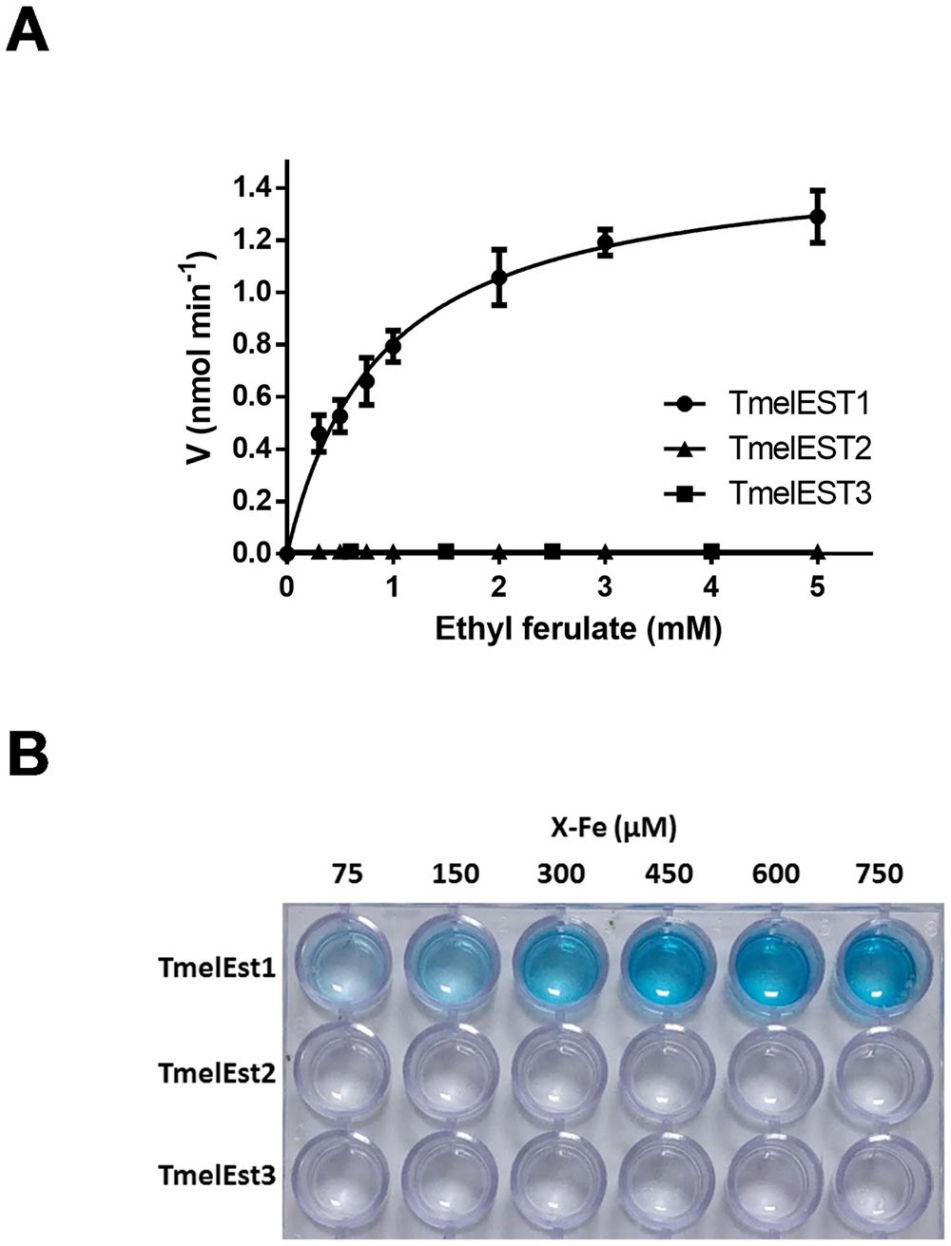
Feruloyl ester hydrolysis by TmelEST1. (**A**) Substrate saturation curve with ethyl ferulate. Increasing concentrations of ethyl ferulate (EFe) were incubated under standard reaction conditions in the presence of a fixed amount (1 µg) of each TmelEST enzyme. EFe hydrolysis and ferulic acid release was measured with a dye-based pH-shift assay (see ‘Methods’ for details). (**B**) Feruloyl esterase activity measured with the cromophoric X-Fe (5-bromo-4-chloroindol-3-yl ferulate) substrate. Reactions (final volume scaled up to 500 µl) were conducted for 60 min at 25 °C in the presence of a fixed amount of each TmelEST enzyme (2.5 µg) and increasing concentrations of the X-Fe substrate as indicated.

Considering the secretory nature of TmelESTs, their preferential expression in mycorrhizae along with multiple GH enzymes but no recognizable feruloyl esterase, and the well-known substrate promiscuity of carboxylesterases, we hypothesized a possible action of the TmelESTs on hydroxycynnamoyl esters and initially tested this hypothesis using ethyl ferulate (EFe) as a model substrate. Only TmelEST1 was capable of hydrolysing ethyl ferulate, with an apparent pH optimum of 8.0 and a Michaelis-Menten substrate saturation curve up to 5 mM EFe (Fig. 3A). The apparent K_m_ derived from this analysis (0.88 mM) is comparable to the K_m_ values previously reported for true microbial feruloyl esterases such as the prototype *Aspergillus niger* enzymes AnFAEA and AnFAEB^35,36^ and the ActOFaeI enzyme from *Actinomyces spp*.^37^, whereas the estimated V_max_ (1.5 U/mg protein) is approximately 10-fold lower.

Similar results were obtained with a chromogenic ferulic acid ester (5-bromo-4-chloroindol-3-yl ferulate; X-Fe)^38^ containing a much bulkier alcohol substituent, whose selective hydrolysis by TmelEST1 was well detectable upon incubation at 25 °C in the presence of X-Fe concentrations as low as 75 µM (Fig. 3B). As previously observed with *p*NPB, also EFe and X-Fe hydrolysis increased by more than 5-fold when the reaction temperature was raised from 25 to 37 °C (not shown).

We also tested 4-nitrophenyl 2-*O*-(*E*)-feruloyl-L-arabinofuranoside and 4-nitrophenyl 5-*O*-(*E*)-feruloyl-L-arabinofuranoside derivatives as potential more complex substrates^39^, but did not detect any hydrolytic activity with either TmelEST enzyme under standard reaction conditions (data not shown).

### Crystal structure of TmelEST2

Because of its superior enzymatic activity and stability, TmelEST2 was selected for crystallization and X-ray diffraction analysis. TmelEST2 crystals (hexagonal space group P6522) contained four protein molecules per asymmetric unit (numbered from A to D), with r.m.s.d. values between Cα atoms of different monomer pairs (referred to monomer A) ranging from 0.18 Å for molecule B to 0.22 Å and 0.29 Å for molecules D and C (Table S3). The higher r.m.s.d. values for monomers C and D are due to the presence of a Triton-X100 molecule derived from the crystallization medium. Although the structures of the four monomers are largely superimposable (see below), the details of the TmelEST2 structure, which was solved at 2.14 Å resolution, are specifically referred to the detergent-free, monomer A molecule.

**Figure 4:**
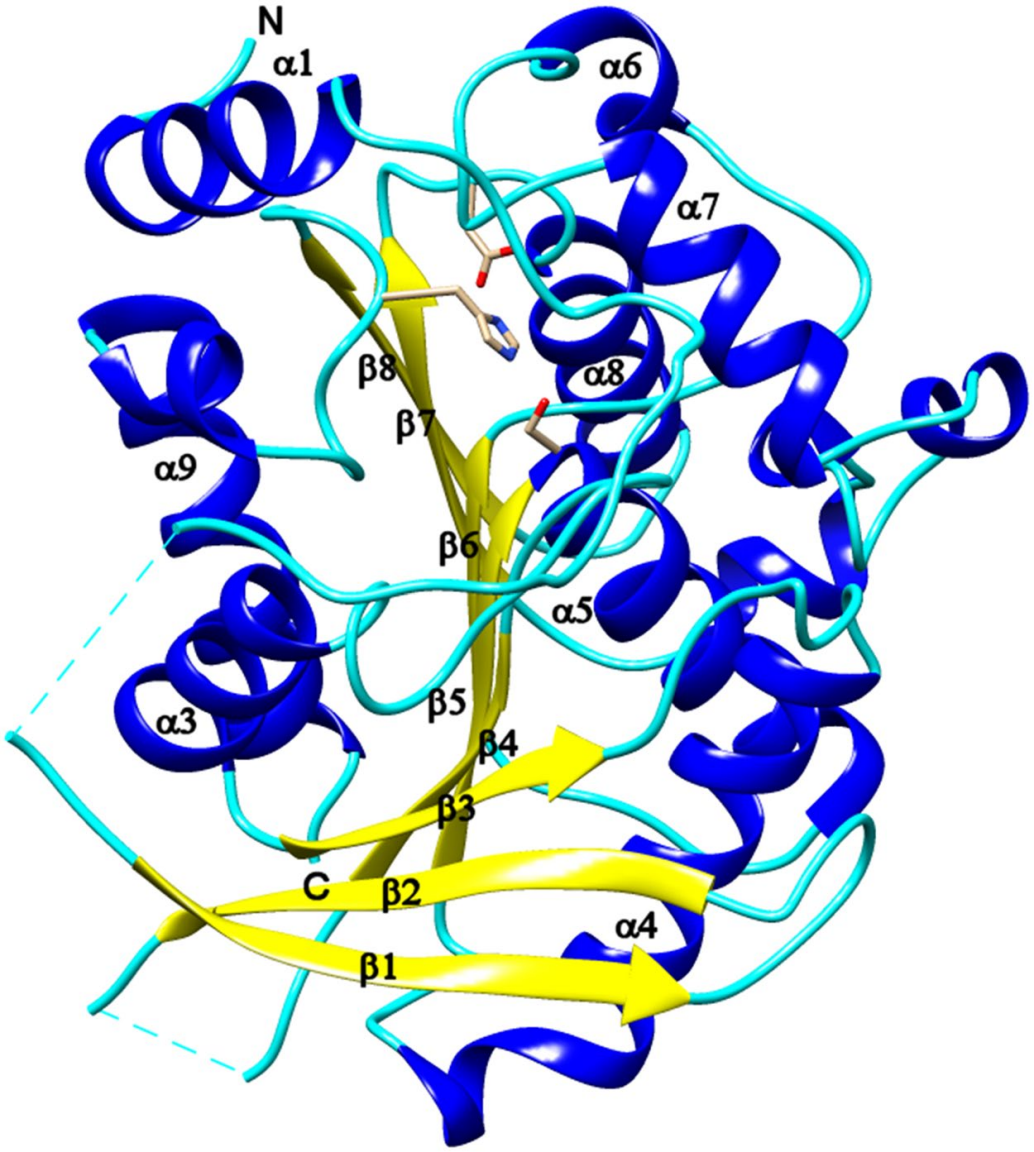
Overall 3D structure of TmelEST2. Ribbon representation of the TmelEST2 structure with the α-helices, β-strands and loops shown in blue, yellow and cyan, respectively. The catalytic Ser-His-Asp triad is rendered as CPK-coloured sticks; the N and C termini of the protein are indicated. The α-helix numbering commonly applied to HSL esterases is utilized for TmelEST2 with the omission of helix α-2 (amino acid residues 57–66), which is unwinded in the *T*. *melanosporum* enzyme. Sequence numbering is referred to the full length protein, including the signal peptide. The structure was solved from residue 34 to 347, with two gaps between positions 67–71 and 100–106.

The 3D structure of TmelEST2 shows an α/β-hydrolase-fold with well discernible cap and catalytic domains (Fig. 4). The latter contains the core region with the catalytic Ser-His-Asp triad, while the less conserved and more dynamic cap domain controls access to the active site and thus likely determines substrate selectivity.

The active site region is a β−sheet consisting of seven parallel strands (β1, β3–8) and one antiparallel strand (β2). The central β−sheet has a left-handed twist orientation and is surrounded by five α-helices: α3 (Arg129-Ser139) and α9 (Leu325-Ala345) on one side; α4 (Val158-Leu177), α5 (Ser190-Lys205) and α8 (Arg290-Lys302) on the other. Amino acid residues 57–66, which in homologous HSL esterases form helix α2, appear to be unwinded in TmelEst2.

The cap domain, which exhibits the highest B-factor, is made of two distinct α−helical regions: α1(Pro37-Ala45),plus the stretch of amino acids corresponding to the missing α2 helix, and helixes α6 (Ala228-Glu232) and α7 (Thr241-Phe251) located in the upper part of the central β-sheet, above the substrate-binding pocket.

Several proteins with significant structural homology to TmelEST2, most of which correspond to the previously identified termophilic bacterial homologs (see Table S2 and Fig. S1), were revealed by a search conducted with the DALI server^40^. The HSL esterases with the highest structural homology are rPPE^18^, RmEstB^20^, PestE^17^ and SshEstI^19^ (Z-scores: 44.6, 40.9, 40.5 and 40.2, respectively; Cα atoms RMSD values: 1.6 Å for 301 residues, 1.9 Å for 297 residues, 1.7 Å for 288 residues and 1.8 Å for 290 residues, respectively). EstE1 derived from a metagenomic sample^28^, AFEst^26^, EST2 from *Alyciclobacillus acidocaldarius*^16^ and RmEstA^20^ also share a significant structural homology with TmelEST2 (Z-scores: 39.8, 39.6, 39.3 and 39.3, respectively; Cα atoms RMSD values: 1.7 Å for 279 residues, 1.8 Å for 291 residues, 2.2 Å for 295 residues and 1.8 Å for 297 residues, respectively. The catalytic core of the proteins with the highest Z-score values is superimposable on the corresponding region of TmelEST2, while there are some differences in the orientation of the cap domain α-helices, as expected from the lower sequence similarity of this region of the protein. In particular, α2 helix unwinding is not observed in any of the HSL homologs of TmelEST2.

### TmelEST2 active site and substrate-binding pocket

**Figure 5:**
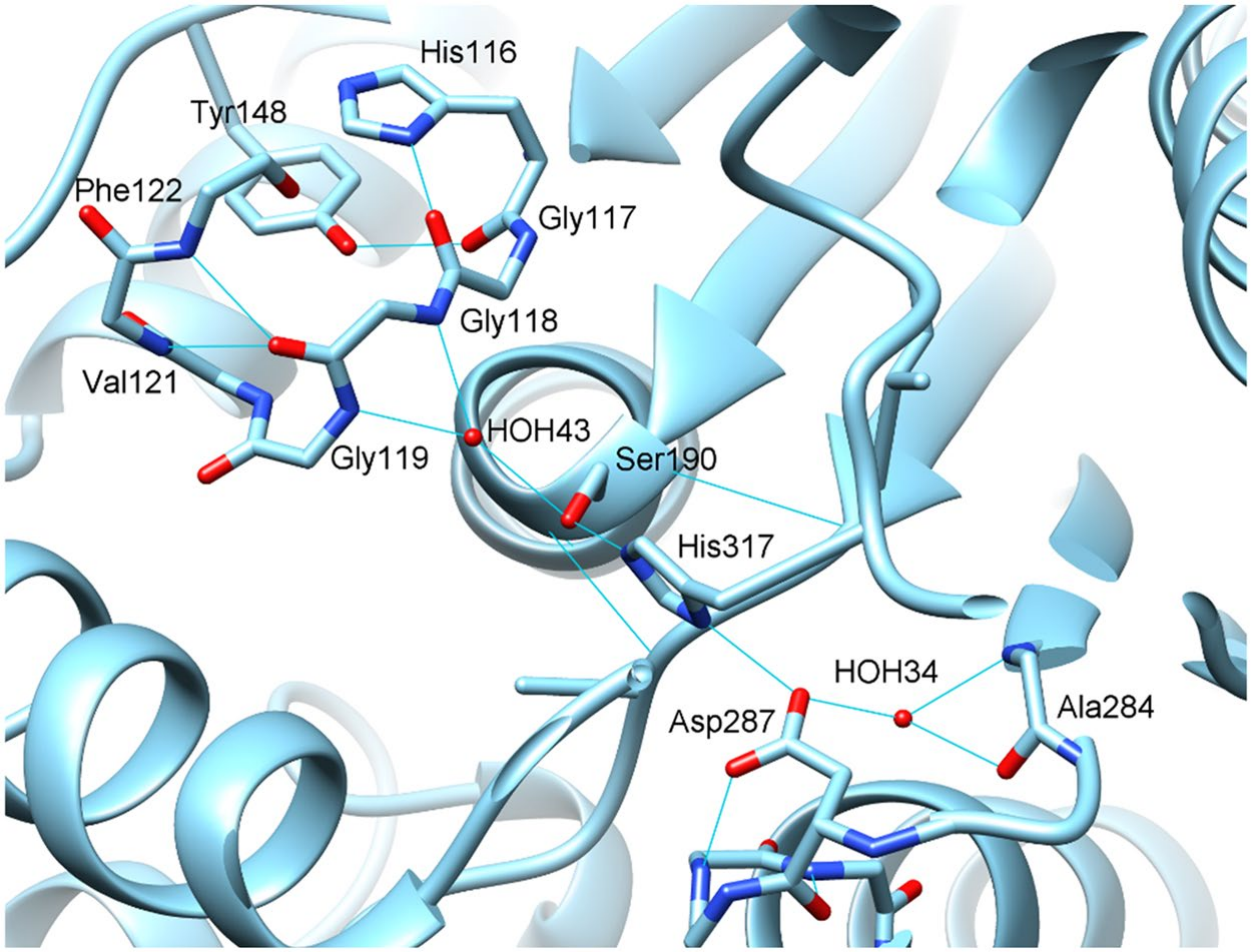
TmelEST2 active site. Active site and oxyanion hole of TmelEST2 rendered in *cyan* ribbon. Key catalytic residues are rendered in stick (CPK-coloured) and labelled; hydrogen bonds are shown as cyan-coloured lines.

The active site of TmelEST2 comprises the Ser190 nucleophile located within the conserved Gly-X-Ser-X-Gly motif, the proton carrier His317 (β7) and the other charge-relay network residue Asp287 (β8). Ser190 is positioned at the end of a hydrophobic funnel and is stabilized by an H-bond between the Oγ atom of the serine hydroxyl and the Nε of His317 (Fig. 5). A H-bond network links the Nδ of His317 to the Oδ2 of Asp287. As in the structures of other α/β-fold hydrolases^16,26,28,41^, oxyanion-hole formation relies on the conserved HGGG motif (His116-Gly119) located upstream to the active site, where His116 is stabilized by H-bonds.

The substrate-binding pocket is composed of two differently sized, tunnel-shaped cavities (overall volume ~1930Å^3^), extending from Ser190 to the outer surface of the protein (Fig. S3A,B). The major cavity (18 Å long) has two entry sites. The internal surface of the major cavity is lined by both hydrophobic and polar residues, while predominantly hydrophobic amino acids line the minor (acyl-binding) cavity.

The Triton X-100 molecule present in monomers C and D is oriented toward the bottom of the substrate-binding pocket (Fig. S4), with its aromatic moiety facing a hydrophobic patch within the major substrate-binding pocket close to Ile244 and Ile289 and to the oxyanion-hole, and the hydrophilic polyethylene oxide chain pointing toward the solvent. The detergent-containing monomer exhibits a closer conformation of the substrate entry site, with the loop regions interposed between residues 48–50 and 56–60 shifted by ~1.7 Å toward the Triton-X100 molecule.

Additional details of the active site architecture were revealed by parallel analysis of a TmelEST2-PMSF structure, showing the sulfonyl fluoride group of the inhibitor covalently bound to the Oγ of Ser190 in all four monomers (Fig. S5A,B). In this structure, the Oα of the PMSF sulfonyl group is hydrogen-bonded to the NH groups of Gly119 and Ala191, while the benzyl group points toward Ala247 within the hydrophobic substrate tunnel. Although PMSF incorporation did not significantly alter the overall structure, by preventing Triton X-100 binding it induced the formation of a more relaxed conformation, mainly due to a rearrangement of the loop (aa. 57–60) surrounding the larger entry site.

### Oligomeric arrangement and the dimer interface of TmelEST2

The four TmelEST2 monomers in the asymmetric unit are arranged as two dimers (A-C and B-D) related by a two-fold axis, one of which (A-C pair) has been used as reference for dimer organization analysis. The dimeric form is stabilized by intermolecular contacts between strands β-8 (hydrophobic interactions and H-bonds) and helices α-9 (salt-bridges); additional, but less extended interactions are centred on helix α-8 (Fig. S6A). All interactions have a centrosymmetric configuration and are directed toward the middle of the interface region. The backbone of the two interacting β-8 strands form four H-bonds: two between the NH and CO groups of Arg311 and two between the same groups of Ile309 and Leu313 (Fig. S6B). Also apparent are interactions between the side chains of Ile 309 and Leu 313, Ile310 and Ile 310, Arg311 and Arg311, plus a salt-bridge network and two H-bonds formed by helix α-9 and the associated loop residues present at the very end of the dimer interface (see Fig. S6C).

Two additional interactions involve Glu285 (β-7/α-8 loop), which forms an intramolecular H-bond with Gly314 and an intermolecular salt-bridge with Arg298 (α-8) (Fig. S6D). This loop, which contains the catalytic Asp287 residue, is further stabilized by the intramolecular hydrophobic interactions formed by Val286 with the cap region residues Phe233 and Leu35. A similar dimerization interface in the mesophilic esterase E25 has recently been shown to be essential for enzyme activity^11^. We therefore investigated by site-directed mutagenesis the intermolecular interactions established by these catalytic triad-proximal residues. Alanine substitution of either Val286 or Arg298 only marginally affected enzyme activity, although the latter mutant (Arg298Ala), in which dimer formation was not affected, exhibited a 9 °C decrease of thermal stability. In contrast, a 70% reduction of enzymatic activity and a similar decrease of thermal stability were caused by Ala substitution of Glu285, both alone and in the context of the Glu285Ala/Arg298Ala double-mutant. However, neither substrate-binding affinity nor preference were affected in either mutant, thus pointing to a predominantly negative effect of Glu285 substitution on the nearby Asp287 catalytic residue, rather than on the more distant substrate-binding site. Another consequence of the Glu285Ala substitution is the disruption of the intramolecular contact with Gly314, which suggests a critical contribution of this interaction to the catalytic efficiency of the enzyme.

### Predicted structures of TmelEST1 and TmelEST3

**Figure 6:**
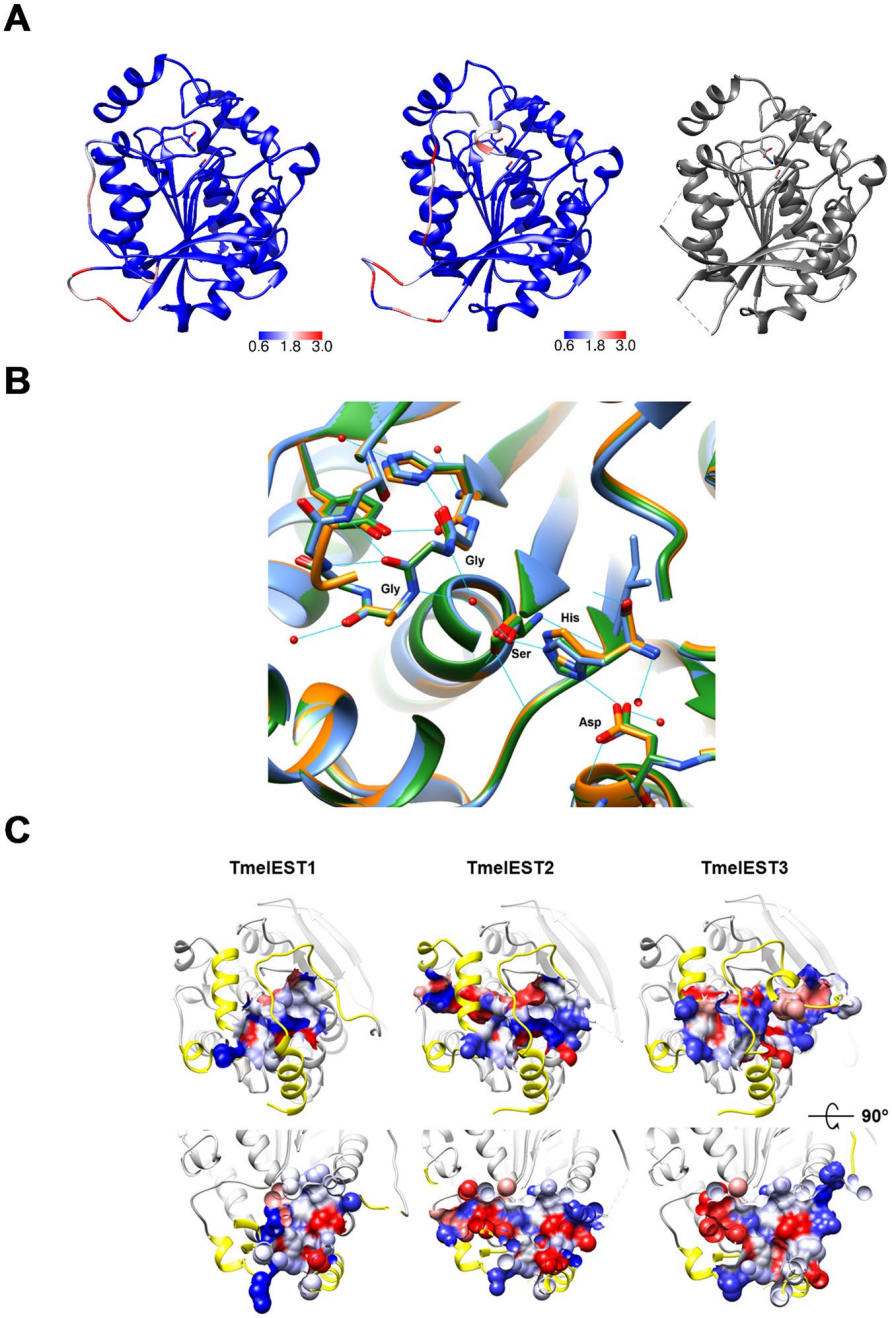
Predicted structures of TmelEST1 and TmelEST3. (**A**) Predicted ribbon structures of TmelEST1 (left) and TmelEST3 (middle) compared with the reference structure of TmelEST2 (right). RMSD values are shown in a blue-red colour scale, with the latter colour corresponding to the highest RMSD values. The TmelEST2 ribbon is in *grey*, with the catalytic triad represented as CPK-coloured sticks. (**B**) Comparative structure analysis of the TmelESTs active site and substrate–binding pockets. The structures of the TmelEST1, 2 and 3 active sites are presented as *orange*, *light-blue* and *green* ribbons, respectively; the side-chains of the catalytic triad and oxyanion hole amino acid residues (individually labelled) of each protein are CPK-coloured; hydrogen-bonds are shown as cyan-coloured lines. (**C**) Cross-section representation of the TmelESTs substrate-binding pockets. The structures of the relevant regions of the three TmelEST proteins are presented as *light-grey* ribbons, with the cap regions highlighted in *yellow*; hydrophobic, charged and polar amino acid residues are shown in *red*, *blue* and *white*, respectively. Protein structures in the lower panel are rotated by 90° with respect to those shown in the upper panel, with a clipping plane drawn in the middle of the substrate-binding pockets.

Based on the structure of TmelEST2, we used the SWISS-MODEL server to predict the structures of the other two *T*. *melanosporum* carboxylesterases. The overall fold of TmelEST1 and 3, including the spatial arrangement of the catalytic triad residues, is essentially identical to that of TmelEST2, with RMSD Cα values of 0.268 Å and 0.228 Å, respectively. As shown in Fig. 6A, the higher RMSD values are associated to the gap (aa. 67–71 and 100–106) and to the unwinded α-2 regions of TmelEST2, where a short α-helix (Ala65-Glu68) is predicted to be present in TmelEST3. Most β-strands residues are unchanged in TmelEST1 and to a lesser extent in TmelEST3, with the majority of substitutions localized to the cap domain and to α-helixes 3 and 9 and associated loops exposed on one side of the protein. Also well conserved are the intermolecular salt-bridges proximal to the catalytic Asp residue (Glu284-Arg297 and Glu291-Arg304 in TmelEST1 and 3, respectively) and the intramolecular hydrophobic interactions between conserved Leu-Val residues that stabilize the α7/α8 loop. Similarly to TmelEST2, the predicted dimer arrangement of TmelEST1 and 3 is also mainly mediated by hydrophobic and H-bond centrosymmetric interactions formed by strand β-8 and helix α-9 (see Fig. S7A–D for details). The main difference is the lack of one salt-bridge in TmelEST1 and two of them in TmelEST3, which might lead to a slightly weakened dimer interface with possible (indirect) effects on the catalytic and thermal stability properties of the two enzymes^11^.

More striking differences were revealed by a comparative electrostatic surface analysis, which indicated the presence in TmelEST2 (pI: 5.70) of a profile considerably less acidic than those of the other two enzymes (Fig. S8A) and similar to the electrostatic surfaces of structurally homologous HSL esterases (Fig. S8B). These are almost completely negative in TmelEST1 (pI: 5.27) and even more so in TmelEST3 (pI: 4.69), with only limited areas of neutral or positive potential present in the cap and the dimer interface regions, plus the bottom of the substrate-binding pocket of TmelEST1. Despite a nearly perfect superposition of the catalytic triad residues, with an essentially identical bonding network for the three enzymes (Fig. 6B), some notable differences are apparent in the substrate-binding region (Fig. 6C), especially within the acyl-binding hydrophobic cavity, which appears to be hindered in TmelEST1 and 50% shorter in TmelEST3 compared to TmelEST2 (8 Å vs. 16 Å). As revealed by CASTp analysis^42^, the estimated volumes of the substrate-binding pockets are 1130Å^3^ and 2254Å^3^ for TmelEst1 and TmelEST3, respectively, compared to 1930Å^3^ for TmelEST2. The slightly larger pocket volume of TmelEST3 compared to TmelEST2 is due to a more open conformation of the pocket entrance.

The narrower acyl-binding pocket of TmelEST1 is due to the obstruction caused by helix α-7, in particular by the side-chain of Arg242, and to a lesser extent by the side chains of Asn243 and Val219. Substitution of Arg242 with alanine did not change substrate selectivity nor affinity significantly, yet caused a two-fold increase of the V_max_ for *p*NPB. Also notable is the presence in the solvent-exposed region of the acyl-binding pocket of TmelEST1 of bulkier hydrophobic amino acids (Val226, Ile254 and Leu250) compared to TmelEST2 (Ala220, Ala248 and Ile244).

## Discussion

An increasing number of putative HSL esterases is being revealed by (meta) genomic sequencing projects but few of them are functionally and structurally characterized, especially enzymes of fungal origin. Taking advantage of an esterase-encoding gene family, a rare occurrence in *T*. *melanosporum*, this work provides a comparative functional and structural characterization of three novel carboxylesterases preferentially expressed in the symbiotic phase of this fungus.

As expected for proteins belonging to the same family, the overall structures of the three TmelEST enzymes are quite similar; they all share the conserved GDSXG consensus motif and well conform to the general α/β-fold structure of HSL carboxylesterases. There are, however, some notable differences in the electrostatic surface profiles, which are considerably more acidic in TmelEST3 and 1 compared to TmelEST2. TmelEST3 has the lowest pI value among structurally similar HSL enzymes including AFest and EST2, and even lower than that of a slightly acidophilic esterase such as EstFa_R (Fig. S8). Despite their different pIs, all TmelESTs display a nearly identical pH optimum, which is in keeping with the data on the pH adaptation and catalytic efficiency reported for the acidophilic EstFa_R and the alkaliphilic SshEstI carboxylesterases^19^. The differences in surface electrostatic profiles among the TmelESTs may thus be aimed at optimizing enzyme interaction with (and activity on) different root districts during mycorrhization.

In most HSL esterases characterized to date, the cap region is highly divergent in both sequence and structure^43^. This is true also for the TmelESTs, in which this region exhibits a somewhat peculiar topology, with the presence of an unwinded helix (α2). The structures of the catalytic domain, including the active site and the oxyanion hole, are, instead, essentially superimposable among the three enzymes and remain essentially unaltered upon PMSF incorporation, at least in the case of TmelEST2. The different kinetic properties of the *Tuber* carboxylesterases are thus likely to be ascribed to other regions of the protein and/or to subtle differences in active site flexibility or protein dynamics.

Perhaps the most notable difference among the TmelESTs is the strikingly smaller volume of the substrate-binding pocket of TmelEST1, which is in line with the size and shape heterogeneity of this part of the protein previously documented for other HSL esterases, including the close structural homologs RPPE and RmEstB (Fig. S9). Instead, the substrate-binding pocket of TmelEST2 is similar in size and electrostatic surface profile to that of PESTE, while the same region of TmelEST3, which has the highest volume, closely resembles that of RmESTA. The shrinkage (and near absence) of the acyl-binding site in TmelEST1 is due to steric hindrance caused by cap domain helix α7 residues and, in particular, by a non-conserved Arg242 residue, whose mutation to Ala led to a two-fold increase of enzymatic activity without any variation of *p*NP-substrate preference or affinity.

Interestingly, while linear kinetics were observed with *p*NP-esters up to C5, all TmelESTs displayed biphasic kinetics with substrates longer than *p*NPV, as previously reported for a thermostable esterase from *Sulfolobus shibatae*^44^. A possible explanation for this peculiar kinetic behaviour, previously proposed for the mutant forms of the EST2^45^ and DSM5389^44^ esterases and supported by our data, is the existence of non-overlapping binding sites for esters bearing acyl-chains of different length^46,47^. Indeed, docking analyses conducted on TmelEST2 confirmed the binding of the short acyl-chains of *p*NPA and *p*NPB into the very interior of the acyl-binding pocket, whereas the opposite orientation (i.e., with the *p*NP group pointing inward) was predicted for *p*NP-octanoate (see Fig. S2C). The latter orientation, which is known to be associated with a marked slow-down of *p*-nitrophenol release, is, in fact, the one we observed for the Triton-X100 molecule bound to TmelEST2. This dual mode of binding would explain the different kinetics observed for *p*NP-esters of different length as well as the lack of a substantial *p*NP-substrate preference by individual TmelESTs.

Even though no significant substrate selectivity was revealed by activity assays using different *p*NP-esters, it is conceivable to imagine that some unique reactivity properties may be associated, at least *in vivo*, to the three TmelESTs. In keeping with this hypothesis is, for example, the unique ability of TmelEST1, whose mRNA exhibits the strongest expression bias for ectomycorrhizae (Table S1), to catalyse feruloyl-ester hydrolysis, which represents an important prerequisite for plant cell wall deconstruction during fungus-plant interaction. In fact, despite the generally non-aggressive nature of ectomycorrhizal fungi compared to phytopathogenic and strictly saprotrophic fungi, symbiosis establishment by *T*. *melanosporum* requires some root cell wall remodelling by dedicated glycoside hydrolases^48^. Genes coding for a limited set of such enzymesbut no recognizable feruloyl esterase gene have indeed been identified in the *T*. *melanosporum* genome^21,34,49^. Feruloyl ester hydrolysis by a HSL esterase has not been reported before, and even structurally very similar enzymes such as TmelEST2 and TmelEST3 are apparently unable to utilize ethyl ferulate or the chromogenic derivative X-Fe as substrates. Indeed, as shown by the docking data in Fig. S10, ethyl ferulate can be accommodated in the acyl-binding cavity of TmelEST2 and 3, but with the ester bond far away from the active site and in an opposite orientation compared to that previously reported for the ferulic acid moiety bound to the prototypic feruloyl esterase FAE_XynZ_^50^. In the case of TmelEST1, instead, ethyl ferulate is predicted to bind into the main substrate-binding pocket in an orientation compatible with nucleophilic attack on the ester bond carbonyl group by the active site Ser residue. TmelEST1 hydrolyzed ethyl ferulate with an apparent K_m_ comparable to that reported for true feruloyl esterases, although with a significantly lower V_max_, but proved inactive on feruloylated glycosyl derivatives. This points to a significant, albeit as yet unexplained difference, between TmelEST1 and true feruoyl esterases, which are structurally related to lipases and capable of interfacial activation. Nonetheless, the evidence that at least some feruloyl-esters can serve as substrates for TmelEST1, together with the broad range of ester compounds acted upon by carboxylesterases and the very occurrence of three distinct symbiosis-induced enzyme forms, leave room for other, as yet unknown substrate and reaction microenvironment specificities. In fact, preliminary data derived from the screening of a small set of potential natural substrates, indicate a weak, but selective activity on the methyl derivatives of the plant hormones methyl salicylate (1.7 µmol/min/mg, *kcat*: 1 s^−1^ estimated K_m_: 0.75 mM) and indole-3-acetic methyl ester (0.67 µmol/min/mg, *kcat*: 0.4 s^−1^ estimated K_m_: 3.35 mM) by TmelEST2 and TmelEST3, respectively.

Initially delineated by molecular phylogenetic and thermal stability analyses, the surprisingly close similarity between the TmelESTs and esterases from hypertermophilic archaea was confirmed by structural data. Particularly revealing, in this regard, is the extended network of intermolecular hydrophobic interactions present at the dimer interface, which represents a hallmark of hyperthermophilic esterases, rarely found in carboxylesterases from mesophilic organisms. Indeed, the higher thermal stability of TmelEST2 compared to the other two TmelESTs correlates with the higher number of hydrophobic and ionic interactions (including the Glu285-Arg298 salt-bridge) that support dimer formation in this particularly stable enzyme form, which closely resembles archaeal HSL esterases such as PestE, EstE1 and AFEst^17,28,51^.

The interactions centered on Glu285 and involved in β-7/α-8 loop-mediated dimer interface formation in TmelEST2 are also present in TmelEST1 and 3, and the residues involved are partially conserved among homologous esterases such as RmEstA and RmEstB. However, at variance with E25, an esterase belonging to the GTASG subfamily whose activity strictly depends on dimer formation^11^, destruction of the intermolecular salt bridge between Glu285 and Arg298 only affects thermal stability in TmelEST2. Instead, the integrity of the intramolecular contacts involving the β-7/α-8 loop residues, especially the hydrogen bond between Glu285 and Gly314, is required to preserve catalytic efficiency. It thus appears as if diverse inter-/intra-molecular contact profiles have been selected during carboxylesterase evolution to achieve the common goal of keeping the loop bearing the catalytic Asp residue in a proper orientation within the catalytic triad.

Considering the likely role of the TmelESTs in mycorrhiza formation, with a delicate balance between root invasion and the establishment of a mutualistic interaction with the plant host, one could speculate that selection of thermophilic enzymes with a highly stable structure, may be instrumental to support adequate levels of enzyme activity even in a harsh environment such as rhizosphere. Particularly intriguing, in this regard, is the possible functional connection between the preferential expression in mycorrhizae of the secreted TmelESTs and of other lipo-hydrolytic enzymes such as the TbSP1 phospholipase^48^ and the recent identification of lipids as key carbon compounds retrieved from the plant in the mutualistic nutrient exchange that takes place at the symbiotic plant-fungus interface^52,53^. Conclusive proof of this hypothesis will require more detailed knowledge on the range of natural substrates that can be acted upon by the secreted TmelEST carboxylesterases in the context of mycorrhiza formation.

A part from their physiological roles, TmelESTs are promising candidates as biotechnologically exploitable enzymes. This is particularly true for TmelEST2, which proved to be the most stable and easy to produce of the three *Tuber* enzymes, with a catalytic efficiency at the very top of all the carboxylesterases that have been characterized so far. The different activities of TmelESTs on non pNP-esters (e.g., feruloyl esters) may also turn out to be advantageous for *in vitro* evolution studies aimed at the isolation of artificial enzyme forms tailored to specific bio-industrial applications.

## Methods

### TmelEST cloning and mutagenesis

*T*. *melanosporum* ECM and FLM cDNA libraries were used for the amplification, respectively, of the TmelEST1 and TmelEST2, and the TmelEST3 coding sequences (with the exclusion of the secretion signal peptide) by touch-down PCR^54^. To allow for directional cloning into a modified pET28 expression vector containing a unique *Cpo*I restriction site, *Cpo*I sequences were included in the oligonucleotides utilized as primers for PCR-amplification (see Table S4). The resulting amplicons were digested with *Cpo*I and ligated into the *Cpo*I-digested and dephosphorylated pET28a-*Cpo* vector, in-frame with an N-terminal 6xHis tag-sequence (see below). Ligation reaction mixtures were transformed into electrocompetent *E*. *coli* DH10T1^R^ cells, followed by selection of positive colonies and identification of individual TmelEST clones by colony-PCR, and transfer of each pET28a-*TmelEST* plasmid into BL21-CodonPlus(DE3)-RIL *E*. *coli* cells for protein expression.

Site-directed mutagenesis was performed with the Q5® Site-Directed Mutagenesis Kit (New England Biolabs) according to the manufacturer’s instructions, using pET28-EST1 and pET28-EST2 as templates and the oligonucleotides listed in Table S1 as mutagenic primers. Reaction mixtures were treated with 2 units of *Dpn*I (37 °C, 30 minutes) and then transformed into *E*. *coli* BL21-CodonPlus(DE3)-RIL cells (Stratagene). Incorporation of the desired mutations was verified by DNA sequencing.

### Expression in *E. coli* and purification of the TmelEST proteins

Overnight cultures (10 ml) of individual TmelEST transformants were transferred to 1 liter of Luria-Bertani (LB) medium and grown to an OD_600_ of 0.6 prior to IPTG (1 mM) addition and a further 12 hrs culture at 20 °C. After sonication in lysis buffer (25 mM Tris-HCl, pH 8.0, plus 0.3 M NaCl, without protease inhibitors)^48^, lysates were centrifuged for 10 min at 9,000 × g and the resulting supernatants were loaded onto HisTrap FF crude columns (GE Healthcare) and eluted with a 20–500 mM imidazole gradient in 25 mM Tris-HCl (pH 8.25) plus 0.5 M NaCl, using an ÄKTA Prime chromatographic system (GE Healthcare). TmelEST-containing fractions, identified by both Coomassie Blue G-250 staining (Bio-Rad) and enzyme activity assays carried out with *p*NPB (see below), were exchanged in 25 mM Tris-HCl (pH 8.5) and adsorbed to anion-exchange chromatography HiTrap-Q FF columns (GE Healthcare), from which TmelESTs were eluted with a 0–0.5 M NaCl gradient. This was followed by removal of the N-terminal His-tag by overnight treatment with thrombin (5U/mg of protein) at 37 °C, and an additional anion-exchange chromatography step as above to remove the cleaved His-tag peptide and the thrombin enzyme. Protein purity was assessed by SDS-PAGE on 11% polyacrylamide gels and by MALDI-TOF mass spectroscopy (Waters-Micromass); protein concentration was determined by UV spectroscopy using A280 molar extinction coefficients of 29005, 27515 and 36120 M-1cm-1 for TmelEST1, 2 and 3, respectively. The same purification procedure was applied to both wild-type and mutant TmelEST forms. A significant loss of enzyme activity upon storage in polypropylene or glass tubes at either 4 °C or −80 °C (likely due to protein adsorption) was prevented by the addition of 1% (v/v) Triton X-100. This detergent, which allowed for complete protein stabilization/solubilization without interfering with enzyme activity, was included in the final buffer (25 mM Tris, pH 8.2, 0.3 M NaCl, 1% Triton X-100), in which the TmelESTs could be stored for months (at 4 °C or frozen) with no loss of protein nor activity.

### Enzyme activity assays

A series of *p*-nitrophenyl (*p*NP) esters with different acyl-chain lengths (C2, C4, C5, C8, C10, C12 and C16) were dissolved in acetonitrile (20–40 mM stock solutions) and used as model substrates. Different concentrations of each *p*NP-ester (0.01–1 mM) were added to the reaction mixtures (25 mM Na-phosphate buffer, pH 7.0, in a final volume of 200 µl at 25 °C) along with different amounts of Triton X-100 stabilized enzyme and *p*NP release was followed spectrophotometrically at 405 nm. Reactions were stopped with the addition of 5 mM PMSF and kinetic parameters were determined by non–linear regression fitting to the Michaelis-Menten equation. Standard reaction mixtures contained 0.4 mM *p*NPB (or *p*NPV) as substrate. Thermal denaturation and temperature dependence assays were performed with a gradient temperature apparatus (Mastercycler, Eppendorf). For thermal denaturation experiments, TmelESTs were incubated at increasing temperatures for 5 min and transferred to an ice-bath for 2 min before measuring residual activity under standard reaction conditions. For the determination of temperature optima, *p*NPB was added to pre-heated reaction mixtures immediately after enzyme addition, followed by a 90 s incubation, reaction blockage with 2 mM PMSF and spectrophotometric quantification of *p*NP release; spontaneous hydrolysis, which only occurred above 60 °C, was measured in parallel enzyme-lacking reactions and subtracted from each temperature data-point. The pH optimum for each enzyme was determined in a pH range comprised between 4.0 and 10.0 using different buffers (sodium acetate, MES, Tris/HCl, CHES) under standard reaction conditions; a correction taking into account spontaneous substrate hydrolysis as a function of pH was applied. Solvent stability was determined for each enzyme by standard assays carried out in the presence of various organic solvents (30% v/v), detergents (5% v/v) or denaturants (2.5 M).

A colorimetric assay (NEFA-HR(2); Wako Chemicals), was used to measure the hydrolysis of medium-chain triacylglycerols (tricaproin, glyceryl trioctanoate and tricaprin), which were solubilized in acetonitrile. Reactions were conducted for 30 min at 37 °C in 25 mM phosphate buffer (pH 7.4), in a final volume of 200 µl containing 4 mM substrate as per NEFA-HR(2) manufacturer’s instructions.

A modified pH-shift assay^31^ was used to measure the hydrolysis of 1-naphthyl acetate, various TAEs (linalyl acetate, tert-butyl acetate, α−terpinyl acetate), the plant esters methyl salicylate, indole-3-acetic methyl ester and methyl jasmonate, and ethyl ferulate. In order to prevent spontaneous hydrolysis, TAEs were dissolved in DMSO and plant methyl esters and EFe in acetonitrile, in the presence of the pH indicator phenol-red (6 µg/ml); organic solvent concentrations in the reaction mixtures (200 µl final volume; 2 mM Tris-HCl pH 8; 5–120 min and up to 24 h for certain compounds at 25 °C) never exceeded 2%. To optimize assay sensitivity, an appropriately low Tris-HCl concentration (0.4–2 mM) was used in all pH-shift assays; no spontaneous substrate hydrolysis was observed up to a 30 mM substrate concentration, even after a 24 h incubation at 25 °C. The final concentration of TAEs and plant methyl esters ranged from 1 to 30 mM, while ethyl ferulate was used at concentrations ranging from 0.25 to 5.0 mM. Reactions were monitored by measuring A560 decrease (red to yellow shift) and quantified by comparing the A_560_ value of the test-sample with that of a reference curve built with the corresponding acids; reaction mixtures lacking either the enzyme or the substrate served as negative controls.

5-bromo-4-chloroindol-3-yl ferulate (X-Fe; a kind gift of Regis Fauré, INSA-LISBP, Tolouse, France) was dissolved in acetonitrile and used in a final concentration range comprised between 0.05 and 1 mM (corresponding to a maximum 2% acetonitrile concentration) with a fixed amount of enzyme (1 µg) under standard reaction conditions^38^. The release of 5-bromo-4-chloro-3-hydroxyindole and its stoichiometric oxidation to the blue-coloured 5 5′-dibromo-4 4′-dichloro-indigo derivative were used to monitor X-Fe hydrolysis (λ_max_ = 615 nm). A quantitative measurement of the reaction, which was completely inhibited by PMSF (5 mM), was prevented by the lack of a suitable standard and by the low solubility of the indigo-derivative product; no spontaneous X-Fe hydrolysis was observed in the absence of enzyme.

4-nitrophenyl 2-*O*-(*E*)-feruloyl-L-arabinofuranoside and 4-nitrophenyl 5-*O*-(*E*)-feruloyl-L-arabinofuranoside (a kind gift of Peter Biely, Institute of Chemistry, Slovak Academy of Sciences, Bratislava, Slovakia)^39^ were dissolved in DMSO (50 mM stock solution) and tested at final concentrations ranging from 0.1 to 1 mM, in the presence of a fixed amount of each TmelEST enzyme (1 µg) in Na-phosphate buffer (pH 7.0) at 25°.

### X-ray diffraction analysis

TmeEST2 crystals were obtained by vapour diffusion in sitting drops (4 µl) containing half-volumes of purified protein (18 mg/ml in 25 mM Tris-HCl pH 8.0, 0.1 M NaCl, 2% v/v Triton X-100) and 20% polyethylene glycol 3350 in 0.2 M KNO_3_ (pH 6.0). An entire diffraction data set (maximum resolution: 2.14 Å) was collected at the PXII beamline of the Synchrotron Light Source of the PSI facility in Villigen (Zurich, Switzerland) using a single crystal of TmelEST2 (space group P6_5_22, unit cell dimensions **a** = **b** = 155.898 Å, **c** = 226.809 Å, 4 monomers/asymmetric unit, corresponding to a V_M_ of 2.62 Å^3^/Da and an approximate solvent content of 53%) and an oscillation of 0.1° for a total of 180°. After indexing and integration with the XDS software^55^, data were merged and scaled with the Scala software^56^. The structure was solved by molecular replacement (Molrep software)^57^ using as template a model generated with the RaptorX server^58^, followed by KoBaMIN^59^ energy minimization. Refinement was performed with the Phenix package^60^ and the resulting model was revised and manually adjusted with the graphic software Coot^61^. The torsion angles simulated annealing option (i.e., non-crystallographic restraints applied to the four monomers) was used for all refinement cycles except the last one. Solvent molecules were added automatically by the Phenix refinement routine. Data collection and refinement statistics are summarized in Table S3. The final crystallographic R factor was 0.1819 (R_free_ 0.2079); the geometrical parameters of the models, verified with the Procheck software^62^, were generally better than those expected for this resolution. PMSF powder was directly added to a crystallization drop containing preformed TmelEST2 crystals, in order to minimize the solvent effect. The structure of the PMSF-containing complex was solved by molecular replacement using the inhibitor-free TmelEST2 structure as template.

### Structure prediction

The three most common 3D structure prediction servers (I-TASSER, RaptorX, SWISS-MODEL) were first comparatively evaluated using TmelEST2 as test protein. The SWISS-MODEL structure homology-modelling server^63^ emerged as the most reliable for side chain orientation, overall intra-molecular bonds, as well as for quality factor parameters and ease of visual inspection. The SWISS-MODEL workspace and the TmelEST2 template were thus used to build the structures of monomeric TmelEST1 (QMEAN6^64^ score: 0.77) and TmelEST3 (QMEAN6 score: 0.745). The predicted structures of the dimeric forms of TmelEST1 (QMEAN6 score: 0.738) and TmelEST3 (QMEAN6 score: 0.714) were generated by superposition of the monomeric structures on the A-C dimer pair of TmelEST2 using the UCSF Chimera software^65^. Reference QMEAN6 scores for the monomeric and dimeric structures of TmelEST2 were 0.841 and 0.797, respectively.

### Other procedures

BLAST searches were conducted against the UniProt (release 2016_08) and PDB databases using the predicted amino acid sequences of the TmelEST polypeptides as queries. SignalP-4^24^ and TargetP 1.1^25^ were used to infer the subcellular localization of the *T*. *melanosporum* carboxylesterases and the presence of a secretion signal peptide. Evolutionary analyses were conducted in MEGA6^66^. Sequence alignments were visualized with GeneDoc^67^; the Neighbor-Joining method^68^ was used for phylogenetic tree construction.

Gel filtration analysis (200 µg of purified TmelEST protein/run in 0.2 ml) was performed with a Superdex 200 HR10/30 column (24 ml; GE Healthcare) equilibrated in 25 mM Tris-HCl (pH 7.5), 150 mM NaCl at a flow-rate of 0.9 ml/min, using an ÄKTA Prime protein purification system. Protein elution was monitored at 280 nm and molecular weights were determined with a calibration curve built with yeast alcohol dehydrogenase (140 kDa), bovine serum albumin (66 kDa) and ovalbumin (44.3 kDa) as molecular mass standards.

For electrostatic potential surface calculations, protein structure files were prepared with the PDB2PQR tool^69^ and analyzed with the Adaptive Poisson-Boltzmann Solver (APBS) interface^70^.

Molecular docking analysis of *p*NPB, *p*NPO and EFe binding was performed with the AutoDockVina program^71^ using default parameters and centering the 40 × 40 × 40 Å^3^ grid spacing box on the substrate binding site of the TmelEST proteins (X: −64.0456, Y: −12.8499, Z: 3.62029). The most favorable ligand-protein docked structures were displayed with the UCSF Chimera software^65^.

## Electronic supplementary material

Supplementary information
