# Author Correction: A family of archaea-like carboxylesterases preferentially expressed in the symbiotic phase of the mycorrhizal fungus *Tuber melanosporum*

**DOI:** 10.1038/s41598-018-29606-0

**Published:** 2018-08-29

**Authors:** Davide Cavazzini, Guido Grossi, Elisabetta Levati, Francesca Vallese, Barbara Montanini, Angelo Bolchi, Giuseppe Zanotti, Simone Ottonello

**Affiliations:** 1 Department of Chemical Life Sciences and Environmental Sustainability, University of Parma, Parco Area delle Scienze 23/A, 43124 Parma, Italy; 2 Department of Biomedical Sciences, University of Padova, Via Ugo Bassi 58/B, Padova, 35131 Italy; 3 Present Address: Interdisciplinary Nanoscience Center (iNANO), Aarhus University, Gustav Wieds Vej 14, 8000 Aarhus, Denmark

Correction to: *Scientific Reports* 10.1038/s41598-017-08007-9, published online 09 August 2017

The original version of this Article contained an error in the title of the paper, where the word “mycorrhizal” was incorrectly given as “mychorrizal”. This has now been corrected in the PDF and HTML versions of the Article, as well as the Supplementary Information that accompanies the Article.

