# Supplementary material for: A family of archaea-like carboxylesterases preferentially expressed in the symbiotic phase of the mycorrhizal fungus Tuber melanosporum
Source: Sci Rep. 2017 Aug 9;7:7628. doi: 10.1038/s41598-017-08007-9 (PMC5550427; doi:10.1038/s41598-017-08007-9)
Supplement: Supplementary file 1 — Supplementary information [file 41598_2017_8007_MOESM1_ESM.pdf]

SUPPLEMENTARY INFORMATION

A family of archaea-like carboxylesterases preferentially expressed in the symbiotic phase of the mycorrhizal fungus *Tuber melanosporum*

Davide Cavazzini[1], Guido Grossi[1§], Elisabetta Levati[1], Francesca Vallese[2], Barbara Montanini[1], Angelo Bolchi[1], Giuseppe Zanotti[2], Simone Ottonello[1*]

[1]Department of Chemical Life Sciences and Environmental Sustainability, University of Parma, Parco Area delle Scienze 23/A, 43124 Parma, Italy. [2]Department of Biomedical Sciences, University of Padua, Via Ugo Bassi 58/B, Padova 35131, Italy.

*Corresponding author: simone.ottonello@unipr.it

[§]Present address: Interdisciplinary Nanoscience Center (iNANO), Aarhus University, Gustav Wieds Vej 14, 8000 Aarhus, Denmark

Supplementary Figures

Figure S1

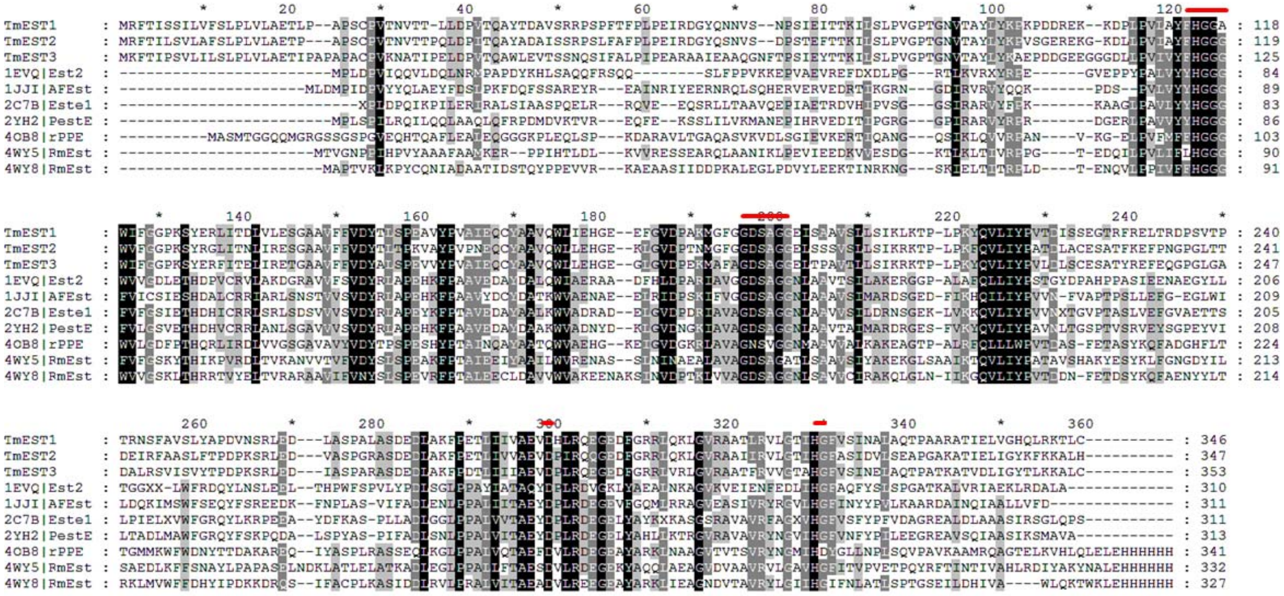

Figure S1. Alignment of the TmelEST sequences with a subset of structural homologs retrieved from the PDB. Conserved amino acids are drawn according to their similarity (*black*, 100% similarity; *dark-grey* >80%; *light-grey*, 50-80%; *white*, <50%). Conserved functional sites are marked with *red* bars.

Figure S2

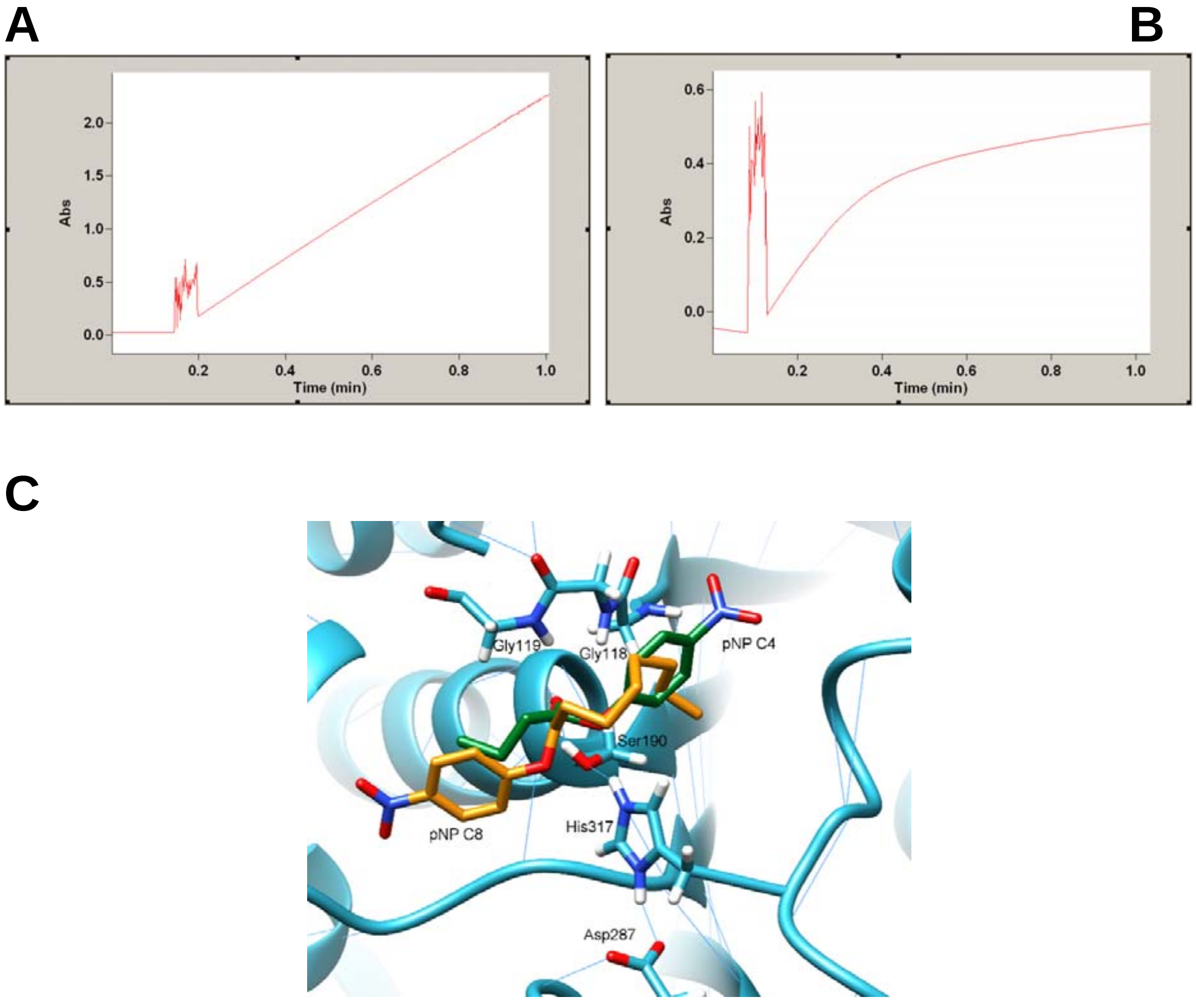

Figure S2. Kinetics of hydrolysis and molecular docking of C4 and C8 *p*NP esters on the structure of TmelEST2. (**A)** Linear rate of hydrolysis of *p*NP-butyrate by TmelEST2. **(B)** Biphasic conversion profile of *p*NP-octanoate. **(C)** Molecular docking modelling of TmelEST2-*p*NPB and TmelEST2-*p*NPO complexes; *p*NP-butyrate and *p*NPoctanoate are shown as CPK-coloured *dark-green* and *orange* sticks, respectively; the catalyitic triad residues (individually labelled) are represented as CPK-coloured *cyan* sticks.

Figure S3

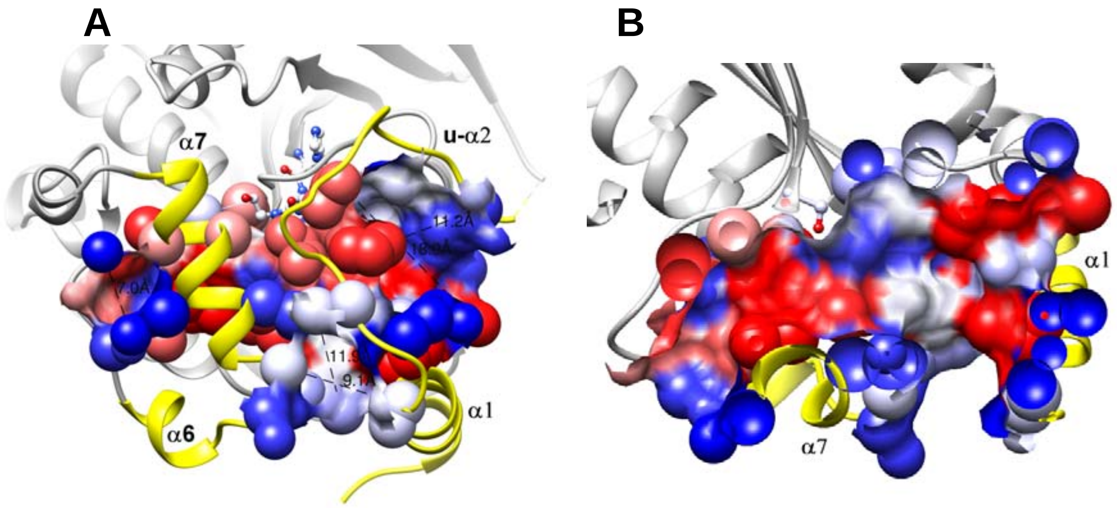

Figure S3. TmelEST2 substrate-binding pocket. (A) Upper view of the TmelEST2 ribbon (light grey) with the cap domain in yellow. Residues lining the hydrophobic pocket are rendered as spheres; surface-exposed hydrophobic residues are shown in red, polar residues in white, charged residues in blue. Oxyanion hole residues are rendered as CPK-coloured balls and sticks. Distances between individual atoms present at the substrate-binding pocket mouth and exit site are labelled in black. **(B)** Cross-section of the TmelEST2 substrate-binding pocket. Ser190 is rendered as a CPK-coloured ball and stick; the other colours and labels are the same as those specified in the legend to panel A.

Figure S4

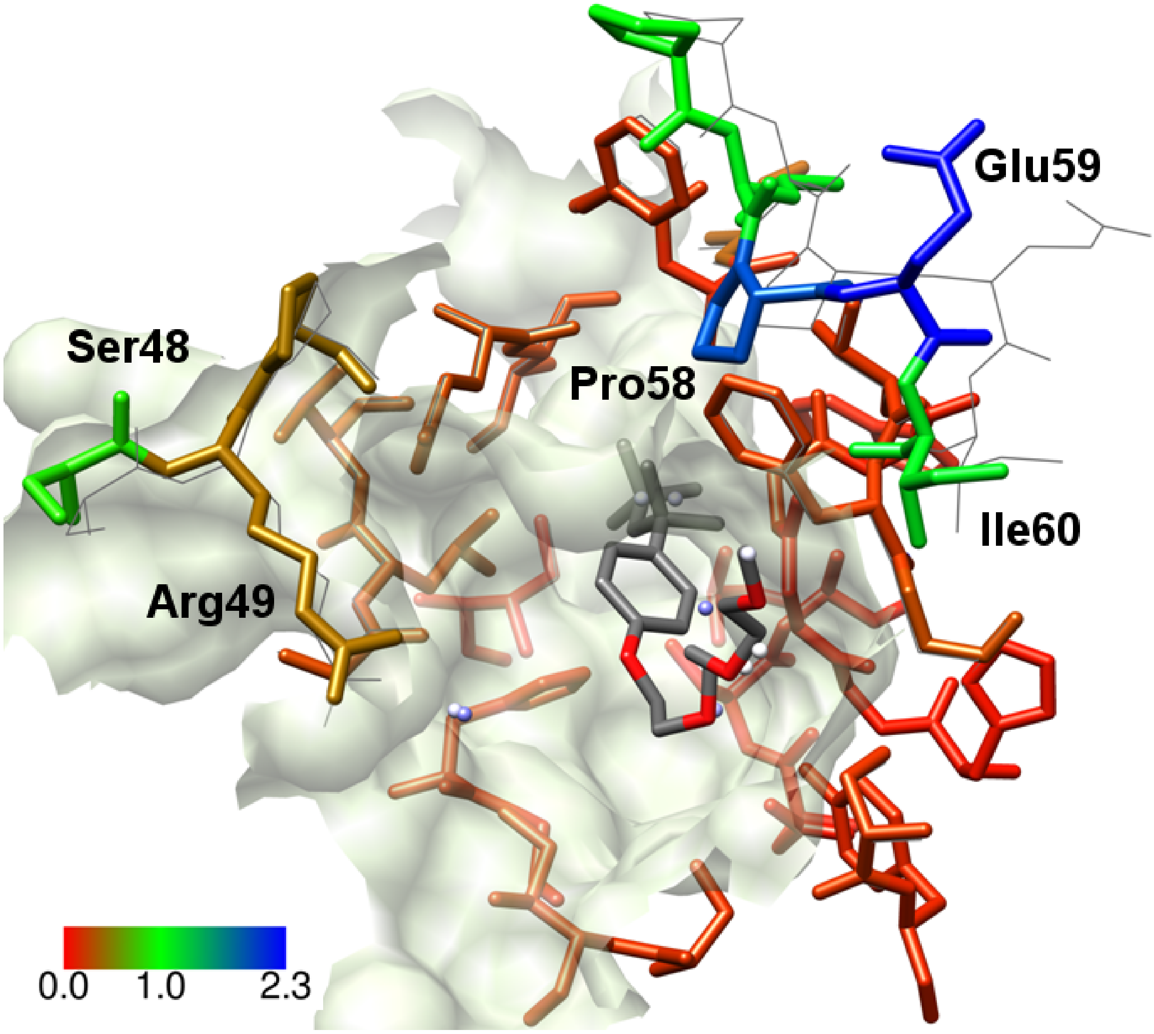

Figure S4. Comparison of the detergent-free and Triton X-100-bound structures of TmelEST2. The TmelEST2-A (Triton X-100-free) and C (Triton X-100-bound) structures of TmelEST2 are superimposed and the amino acid residues close to the Triton-X100 molecule are highlighted. TmelEST2-A residues are rendered as *dark-grey* wires, while TmelEST2-C residues are rendered as sticks drawn according to a rainbow-scale distance (Å) color-code with the higher RMSD values in blue. The Triton X-100 molecule is rendered in CPK-coloured *dark-grey* sticks; the transparent surface area of the substrate-binding pocket is shown in *light-grey*.

Figure S5

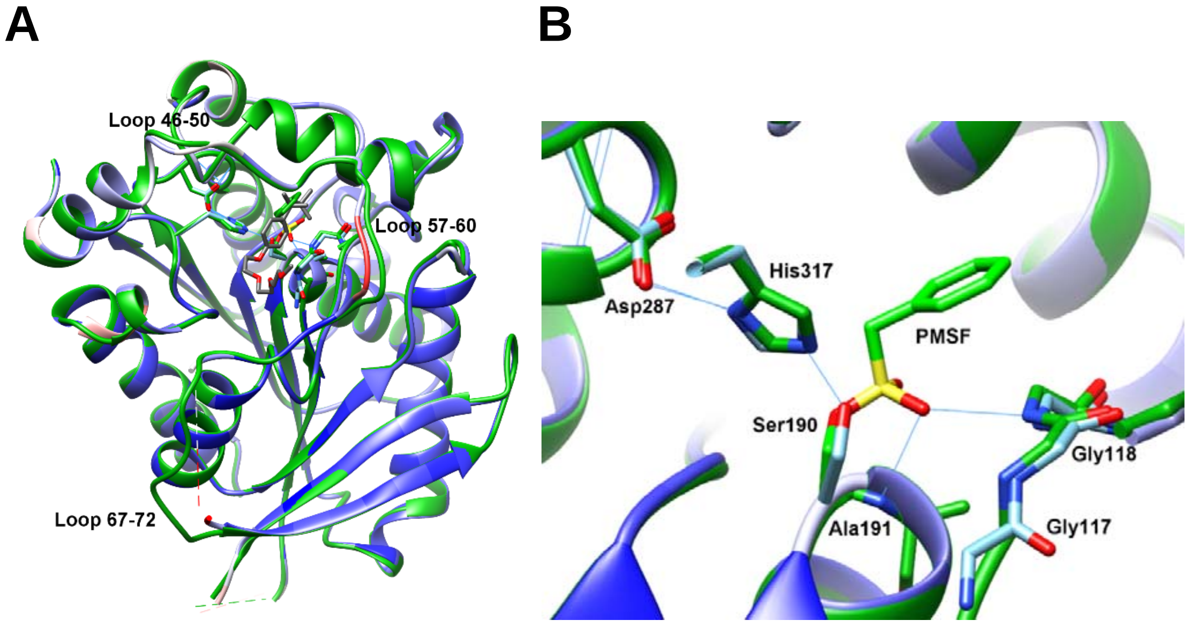

Figure S5. Structure of the TmelEST2-PMSF complex. **(A)** Ribbon structure of the TmelEST2 (monomer D)-PMSF complex (*green*) superimposed onto the native (PMSF-free) structure of TmelEST2 (monomer D; shown in a *red to blue* colour scale, with the higher RMSD regions in *red*). The catalytic triad and oxyanion-hole amino acid residues are rendered as CPK-coloured *green* and *cyan* sticks for the PMSF-bound and PMSF-free structures, respectively; the Triton X-100 molecule is shown in *dark-grey*. **(B)** Close-up of the active site superposition of the PMSF-free and PMSF-bound structures of TmelEST2 coloured as in (A). Hydrogen bonds are depicted as *cyan*-coloured lines; the catalytic triad and oxyanion-hole residues are rendered as CPK-coloured *green* and *cyan* sticks for the PMSF-bound and PMSF-free structures, respectively. R.m.s.d. values ranging from 0.244Å to 0.339Å were obtained from the superposition of equivalent Cα atoms in individual TmelEST2 monomers.

Figure S6

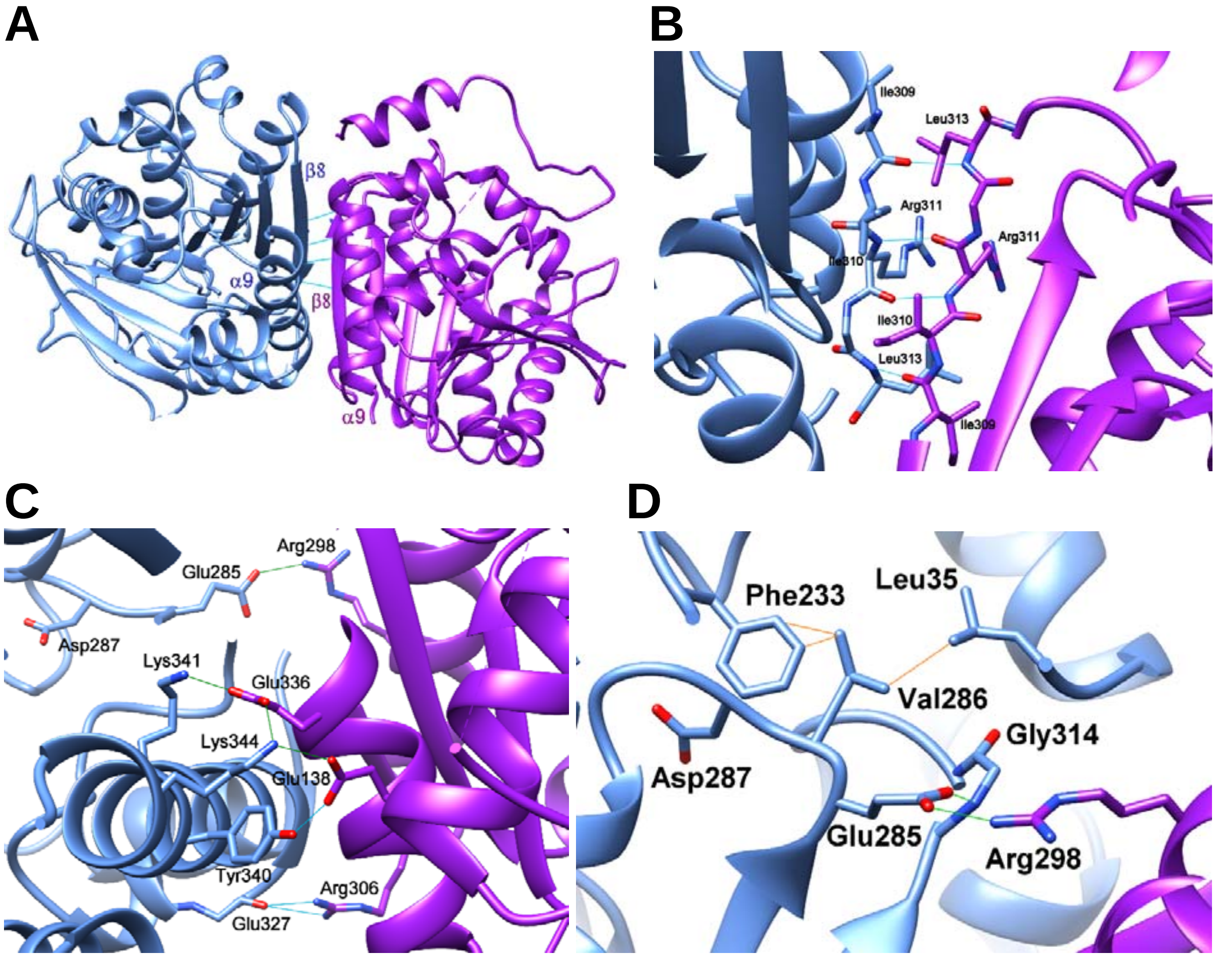

Figure S6. TmelEST2 dimer interface. **(A)** Hydrogen bonds present at the dimer interface of TmelEST2 (A-C monomers; rendered in ribbon, with monomer A in cyan and monomer C in violet). The hydrogen bonds formed by anti-parallel backbones residues belonging to β-8 are shown as cyan-coloured lines. **(B)** TmelEST2 dimer interface contacts formed by β-8 residues (ribbon rendering, with monomers A and B in cyan and violet, respectively, as in panel A). Hydrogen bonds are depicted as cyan-coloured lines; the side-chains of amino acid residues involved in hydrophobic interaction contacts are represented as CPK-coloured sticks and labelled. **(C)** Salt bridges and hydrogen bonds at the dimer interface (ribbon rendering, with monomers A and B in cyan and violet, respectively, as in panel A); the side-chains of the involved amino acid residues are

represented as CPK-coloured sticks and labelled. Ionic bonds and hydrogen bonds are shown in green and cyan, respectively; part of the ribbon structure is omitted for clarity. **(D)** TmelEST2 dimer interface bond network near to the catalytic Asp residue; contact-forming amino acid residues are depicted as CPK-coloured sticks. Individual TmelEST2 monomers are shown as cyan or purple ribbons as above; hydrogen bonds and salt bridges are depicted as green lines; hydrophobic contacts are shown as orange-coloured lines.

Figure S7

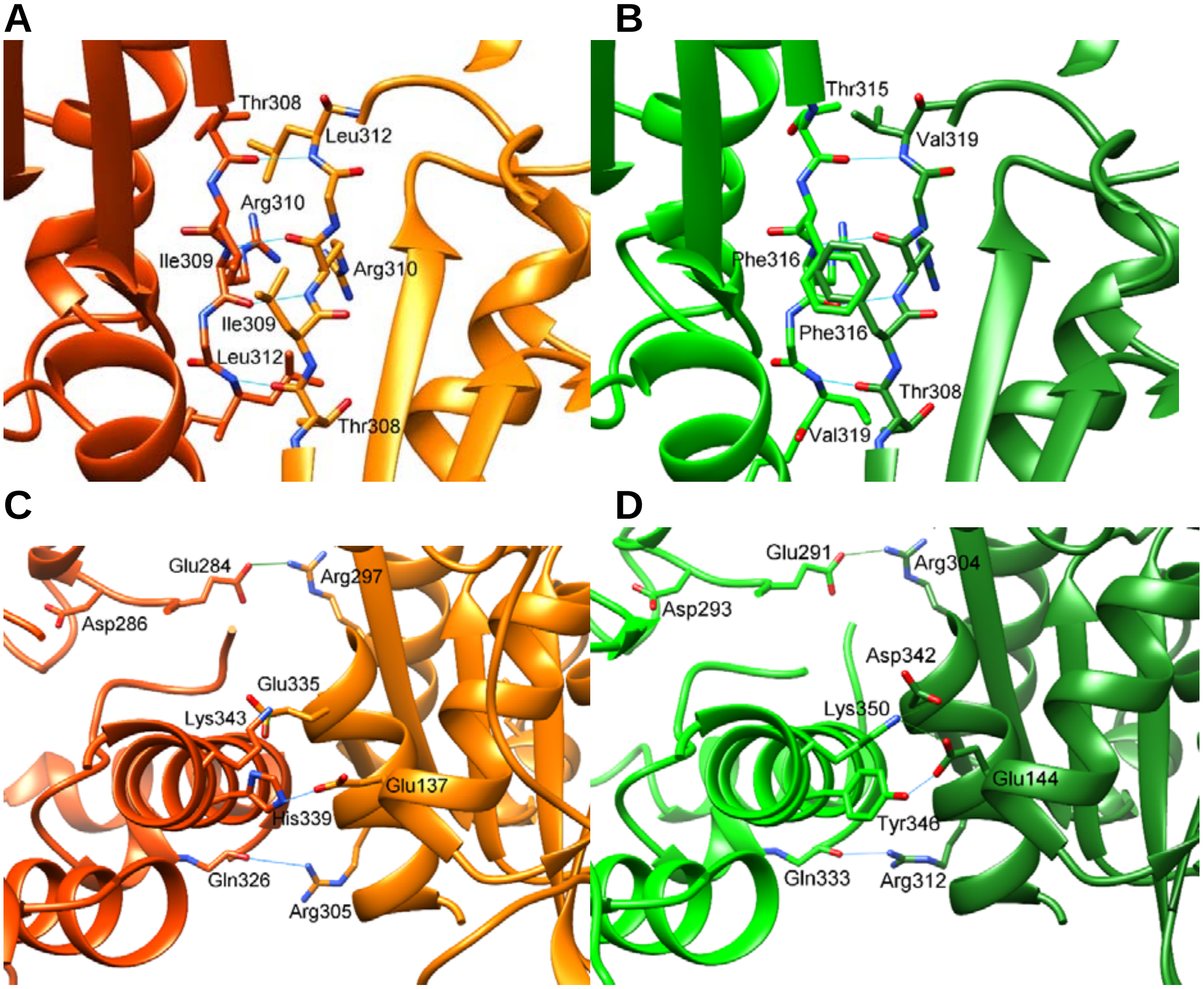

Figure S7. Dimer interface interactions of TmelEST1 and TmelEST3. (A) Ribbon representation of the hydrogen-bonds formed by strand β-8 residues at the TmelEST1 dimer interface; individual monomers are depicted as *red* and *orange* ribbons. **(B)** Same as (A) for TmelEST3, with individual monomers represented as *light-green* and *green* ribbons. In both panels the side-chains of the amino acids (individually labelled) involved in the dimer interaction are represented as sticks; ionic bonds are depicted as *green* lines, hydrogen-bonds as *cyan* dotted lines. The side-chains of amino acids (individually labelled) involved in hydrophobic interactions are represented as sticks; part of the ribbon structure is omitted for clarity. **(C)** Salt-bridges and hydrogen-bonds formed at the dimer interface of TmelEST1 outside of the strand β-8 region. **(D)** Same as (C) for TmelEST3. Colour codes are the same as in panels (A) and (B); part of the ribbon

structure is omitted for clarity. The strands β-8 H-bond network (a total of four H-bonds) predicted for TmelEST1 (two H-bonds between Thr308 and Leu312 and two between the side-chains of Arg317) and TmelEST3 (Thr315/Leu319 H-bonds plus two H-bonds formed by the side-chains of Arg317) is nearly identical to that of TmelEST2.

In the three enzymes the H-bond network is stabilized by hydrophobic interactions: Ile 309-, Leu312-, Thr308- and Arg310-mediated interactions in TmelEST1; Phe316-, Thr31-5 and Val319-mediated interactions, but no Arg317-mediated interaction in TmelEST3. As in the case of TmelEST2, additional polar interactions at the dimer interface (but outside of the central anti-parallel β-8 strand region) are predicted by the Pisa server[1]. These mainly involve amino acid residues located on helix α-9 (**see Fig. S7 A, B and Fig. S6C**), with a salt-bridge between Glu335 and Lys343 plus two H-bonds between Nε2His339 and OεGlu137 and between the carbonyl group of Gln326 with the amino group of Arg305 in TmelEST1; and a salt-bridge network centred on Asp342, Lys350 and Glu144 residues, plus two H-bonds formed by the Tyr346 hydroxyl and OεGlu140 and by the carbonyl group of Gln333 with the amino group of Arg312 in the case of TmelEST3.

Figure S8

A

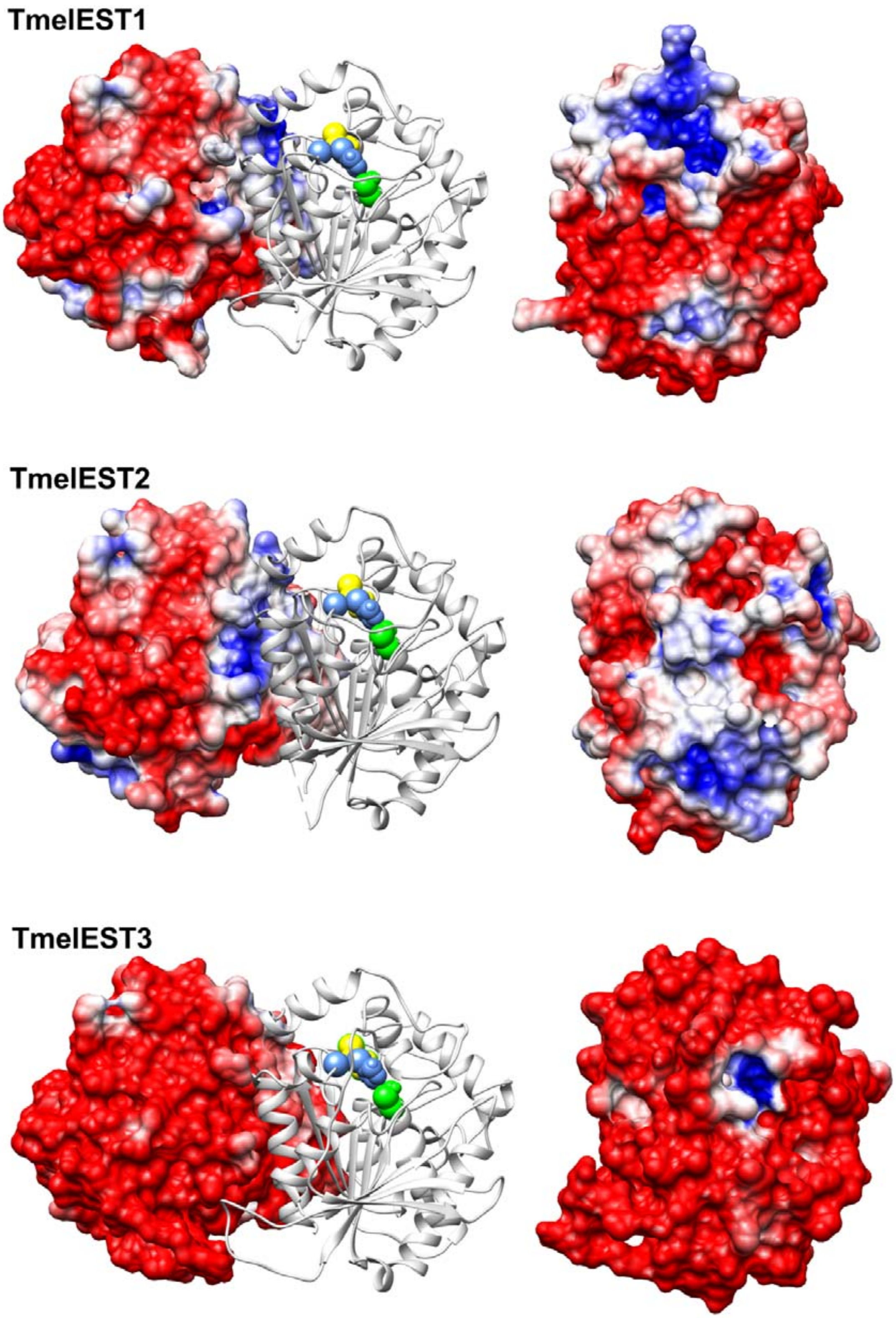

B

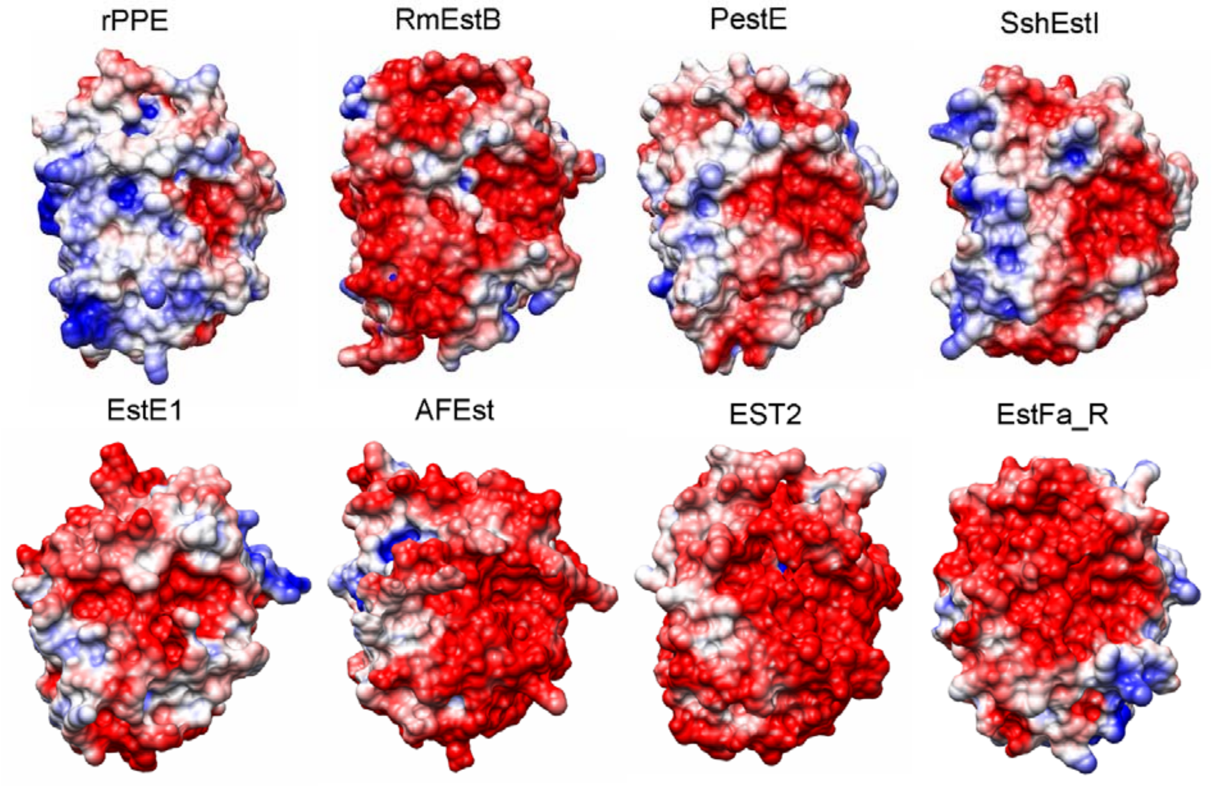

Figure S8. Electrostatic surface potential profiles of the TmelESTs and their closest structural homologs (A) TmelEST electrostatic surface potentials: the accessible surfaces of each protein are rendered in a *red* (-3kT, maximum negative value) to a *blue* (+3kT, maximum positive value) colour gradient according to their electrostatic potentials calculated with APBS. The surface potentials of the dimer interface region are shown on the *left-side*, with one monomer represented as a *light-grey* ribbon and amino acid residues forming the catalytic triad represented as spheres (Ser in *green*, His in *blue* and Asp in *yellow*). The electrostatic potential surfaces of the monomers, presented as ribbons and rotated by 180° with respect to the structures presented in the *left-side* panels, are shown on the *right*. **(B)** Electrostatic surface potentials of a selected subset of TmelEST structural homologs. The structures of the homologous carboxyl-esterases are oriented in the same way as the reference TmelEST2 structure shown in the *right-side* part of panel (A). The accessible

surfaces of each protein are rendered in a *red* to *blue* (+3kT, maximum positive value) colour gradient as in (A). The source microorganisms of the carboxyl-esterases utilized for this comparison are the following: rPPE, *Pseudomonas putida*[2]; RmEstB, *Rhizomucor miehei*[3]; PestE, *Pyrobaculum calidifontis*[4]; SshEstI, *Sulfolobus shibatae*[5]; EstE1, metagenomic sample[6]; AFEst, *Archeoglobus fulgidus*[7]; EST2, *Alyciclobacillus acidocaldarius*[8]; RmEstA, *Rhizomucor miehei*[3].

Figure S9

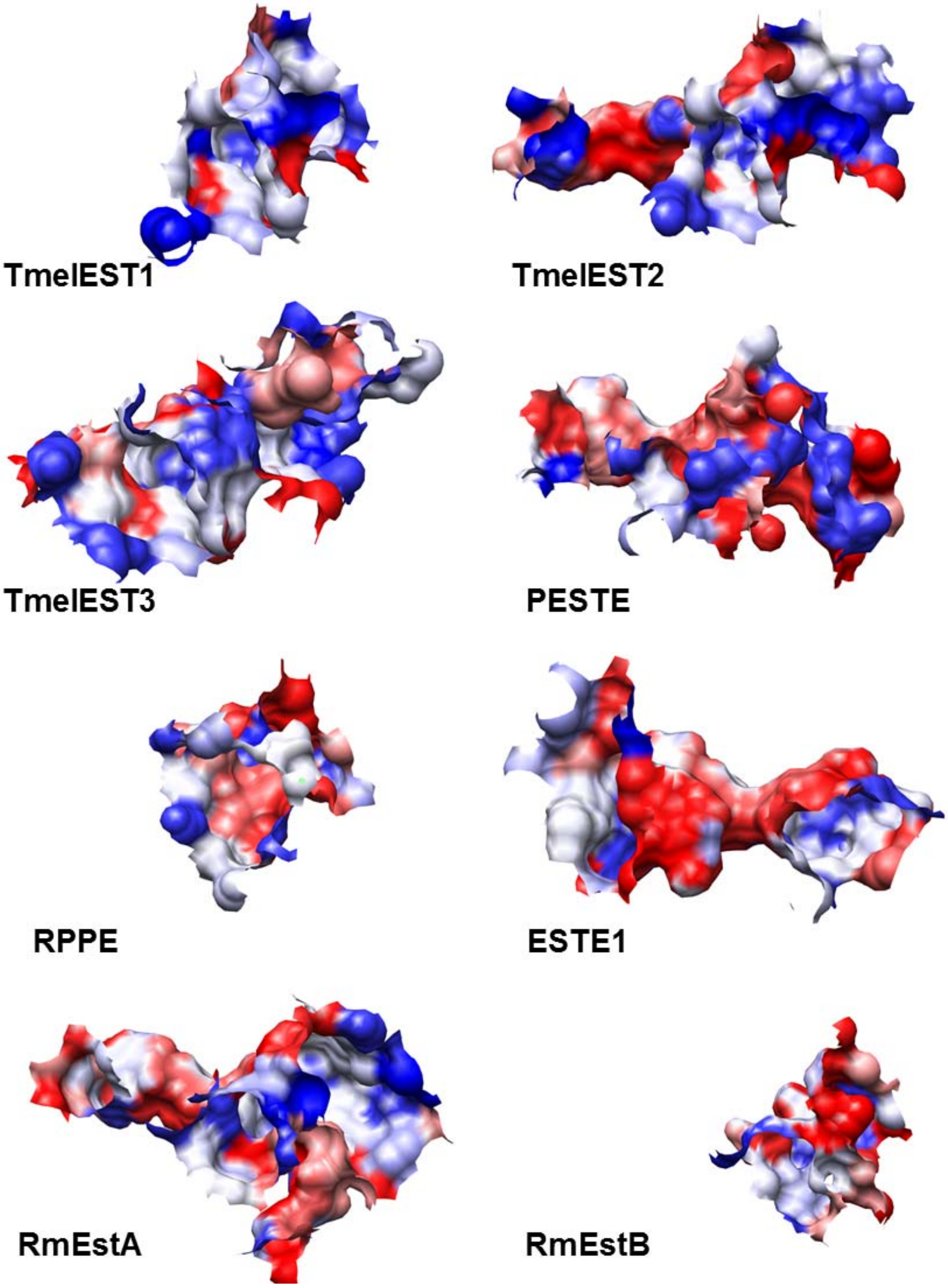
TmelEST1
TmelEST2
TmelEST3
PESTE
RPPE
ESTE1
RmEstA
RmEstB

Figure S9. Comparison of the active site regions of various HSL esterases structurally related to the TmelEST enzymes. Hydrophobic surfaces are shown in *red*, polar surfaces in *white* and charged surfaces in *blue*.

Figure S10

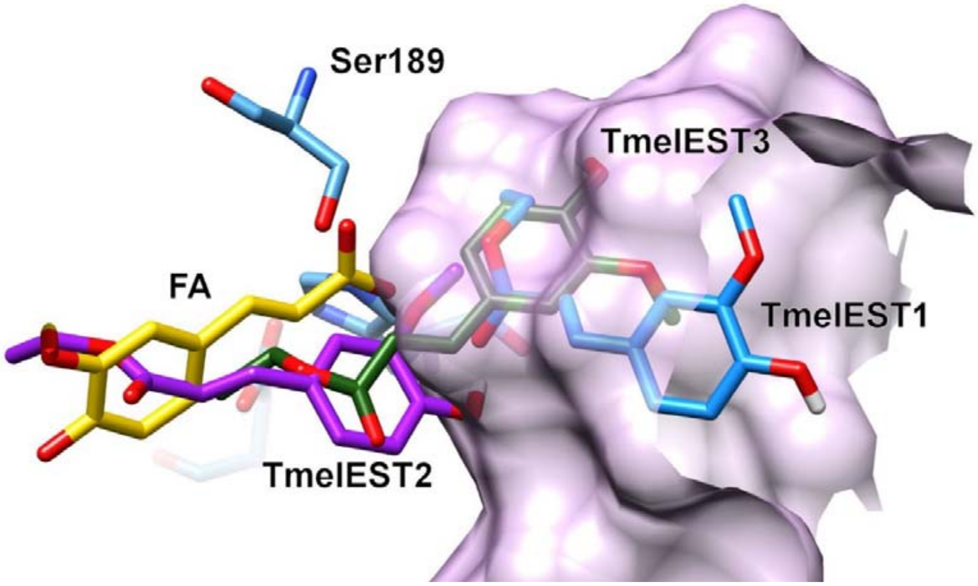

Figure S10. TmelEST1 feruloyl ester docking analysis. Molecular docking analysis of the interaction of ethyl ferulate with the substrate-binding pocket of the *Tuber* esterases. The results are presented as superimposed TmelEST structures showing the lowest energy conformations predicted for each enzyme-EFe complex. The surface of the substrate-binding pocket, with the catalytic Ser CPK/cyan colored, is only shown for TmelEST1. Ethyl ferulate is rendered in CPK-coloured *cyan*, *purple* and *green* sticks, for TmelEST1, TmelEST2 and TmelEST3, respectively. The ferulic acid (*FA*) moiety interacting with the active site region of the feruloyl esterase FAE_{XynZ} from *Clostridium thermocellum*[9] is shown by comparison and CPK-coloured in *yellow*.

Supplementary Tables

Table S1. Expression profiles of the TmelEST mRNAs in different stages of the *T. melanosporum* life-cycle[1]

	RNA-seq				Array			
	**RPKM[1] FLM[2]**	**RPKM ECM[2]**	**RPKM FB[2]**	**ratio ECM/FLM**	**FLM**	**ECM**	**FB**	**ratio ECM/FLM**
TmelEST1	0.0	98.9	1.2	**>99**	2.0	19542.5	179.8	**9795.7***
TmelEST2	42.4	60.8	7.3	**1.4**	1882.7	3924.4	1636.9	**2.1**
TmelEST3	7.1	119.4	4.2	**16.8**	781.8	4531.6	782.4	**5.8***

[1]Expression data, derived from two independent transcriptome studies, were extracted from Tisserant et al (2011)[10] and Martin et al (2010)[11] for RNA sequencing (RNA-seq) and oligoarray analyses, respectively; RNA-seq data are expressed as Reads Per Kilobase per Million mapped reads (RPKM).

[2] FLM: free-living mycelium; ECM: ectomycorrhizae; FB: fruiting-body.

* p-value < 0.01

Table S2. Sequence identity between the TmelESTs and their structural homologs retrieved from the PDB[1]

	**AFEst[2]**	**Est2**	**EstE1**	**PestE**	**RmEstA**	**RmEstB**	**rPPE**
**TmelEST1**	37	32	35	35	34	37	41
**TmelEST2**	37	35	33	32	31	33	39
**TmelEST3**	36	33	33	39	37	40	42

[1]Percent identity values derived from BLAST-P analysis are reported.

[2]The source microorganisms of the carboxyl-esterases utilized for this comparison are the following: AFEst, *Archeoglobus* fulgidus[7]; EST2, *Alyciclobacillus acidocaldarius*[8]; EstE1, metagenomic sample[6]; PestE, *Pyrobaculum calidifontis*[4]; RmEstA, *Rhizomucor miehei*[3] RmEstB, *Rhizomucor miehei*[3]; rPPE *Pseudomonas putida*[2].

Table S3. Data collection and refinement statistics

	**TmelEST2**	**TmelEST2-PMSF**
Wavelength (Å)	0.99988	0.97623
Space group	$P6_522$	$P6_522$
Cell dimensions		
a, ***b***, ***c*** (Å), Z	**a**=**b**=158.898, **c**=226.809, Z=4	**a**=**b**=157.931, **c**=231.410, Z=4
Resolution (Å) *	47.28 – 2.14 (2.26 – 2.14)	47.20 - 2.37 (2.45-2.37)
R_{merge}	0.094 (0.711)	0.172 (0.74)
R_{pim}	0.031 (0.239)	0.042 (0.382)
Unique reflections	92970 (13249)	68933 (6269)
$\langle I/\sigma(I)\rangle$	71.46 (3.3)	11.8 (2.0)
Completeness (%)	99.8 (98.7)	99.4 (94.3)
Multiplicity	11.1 (10.6)	18.3 (10.5)
**Refinement**		
R_{work} / R_{free}	0.1819 / 0.2077	0.1967 / 0.2449
No. protein atoms	9227	9396
Ligands/solvent	50/692	0 / 491
Average B factor	43.6	46.7
R.m.s. deviations		
Bond lengths (Å)	0.008	0.008
Bond angles (°)	1.16	1.15
**Validation statistics**		
Ramachandran favoured (%)	98.1%	95.4%
Allowed	1.9%	4.4%
Outliers	0.0%	0.6%
Rotamer outliers	0.7%	1.6%
C-beta outliers	0	0
Overall score	1.36	1.79
PDB code	5MIF	5MII

* Values in parentheses refer to the last resolution shell.

Table S4. Oligonucleotide primers utilized in this study

Name	Sequence (5'-3')
**pET28-*Cpo*I cloning**	
TmelEST1-F	CCC GGT CCG GAAACCCTCCCCGCTCCC
TmelEST1-R	CACGGACCGCTAGCACAAGGTCTTCCTCAACTGAT
TmelEST2-F	TAAATATAAA C GGT CCG GAAACCCCCGCTCCCTCC
TmelEST2-R	AATTATTTTA CGG ACC G CTAGTGCAAGGCTTTCTTGAAC
TmelEST3-F	CCC GGT CCG GAAACCATCCCCGCTCCC
TmelEST3-R	CACGGACCGCTAGCACAAGGCCTTCTTCAACGTA
**pPIC-Z cloning**	
TmelEST2_XhoI-F	TAAATATAAA CTCGAG AAAAGA GAG GCT GAA ACC CCCGCTCCCTCC
TmelEST2_XhoI-R	AATTATTTTA CTCGAGCTAGTGCAAGGCTTTCTTGAAC
**Site-directed mutagenesis**	
TmelEST1-R242A_F	TACCCCCACCGCTAACAGCTTCGC
TmelEST1-R242A_R	ACACTGGGGTCTCGAGTG
TmelEST2-R298A _F	GGATTTTGGAGCTAGGTTGCAGAAGTTGG
TmelEST2-R298A_R	TCGCCCTGCTGTCTAATC
TmelEST2-E285A _F	AGTCGTCGCAGCTGTGGATCCGATTAG
TmelEST2-E285A_R	ATGAGGGTTTCCGGGAAC
TmelEST2-V286A _F	CGTCGCAGAAGCTGATCCGATTAG
TmelEST2-V286A_R	ACTATGAGGGTTTCCGGG

Supplementary Methods

TmelEST2 production in *P. pastoris*

Following PCR amplification using pET28-TmelEST2 as template and the *Xho*I site-containing oligonucleotides TmelEST2_XhoI-F and TmelEST2_XhoI-R as primers (see Table S3), the TmelEST2 amplicon was digested with *Xho*I and ligated to the shuttle vector pPICZα-Δ under control of the AOX1 promoter and in-frame with the sequence coding for the prepro-α factor secretion signal peptide. The ligation reaction mixture was transformed and propagated in *E. coli* DH10T1^R cells under zeocin (100 μg/ml) selection conditions. Transformants harbouring pPICZα-TmelEST2 plasmids with a correctly oriented TmelEST2 insert were first identified by colony-PCR. After sequence verification and amplification in *E. coli*, a selected pPICZα-TmelEST2 plasmid was linearized by *Sac*I digestion (the only unique restriction site present upstream to the AOX1 promoter) and 10 μg of plasmid were transferred into *P. pastoris* (KM71H strain) by electroporation[12].

Transformed *P. pastoris* cells were grown on Yeast Extract Peptone Dextrose Medium (YPDS) plates in the presence of increasing concentrations of zeocin (up to 500 μg/ml), in order to select for multiple-integration transformants (EasySelect Pichia Expression Kit Version G.2005 Instruction Manual; Invitrogen). After verification of TmelEST2 insertion in the *P. pastoris* genome by colony-PCR using the TmelEST2_XhoI-F and TmelEST2_XhoI-R primers (Table S4), a single colony of a *P. pastoris* transformant resistant to the highest zeocin concentration was inoculated into 1 liter of Buffered Glycerol-complex Medium (BMGY). Following overnight growth at 30°C, cells were harvested, washed with sterilized water, and resuspended in 100 ml of Buffered Methanol-complex Medium (BMMY) at room temperature. TmelEST2 expression was induced for 5-days with daily 0.5% methanol additions. In order to maximize protein production, a previously described multi-cycle induction protocol was employed[12]. Briefly, at the end of the fifth day of culture under

methanol-induction conditions, the culture medium was collected by centrifugation at 3000 x g for 5 min, yeast cells were resuspended in 100 ml of fresh 2% methanol-containing BMMY and cultured for five days without any further methanol addition. The conditioned media derived from four sequential induction cycles were pooled and tested for TmelEST2 secretory production by SDS-PAGE and enzyme activity assays, which revealed a high (>75%) TmelEST2 enrichment in the culture medium. The pooled media were then subjected to further purification by cold-acetone precipitation, with the addition of 3 volumes of ice-cold acetone, followed by 2 min vortexing, 60 min incubation at -20°C and recovery of the precipitated protein by centrifugation for 10 min at 14,000 x g. After acetone evaporation at 25°C and resuspension of the pellet in 10 ml of 2 M $(NH_4)_2SO_4$ in 25 mM Tris-HCl (pH 7.0), the protein was loaded onto a phenyl-HiTrap column and eluted with a 2 M $(NH_4)_2SO_4$ - 25 mM Tris-HCl (pH 8.0) gradient. TmelEST2-containing fractions were pooled and exchanged in 25 mM Tris-HCl (pH 7.0) buffer supplemented with 1% (v/v) Triton X-100. One liter of starting culture subjected to four sequential induction cycles yielded approximately 140 mg of homogenously purified TmelEST2. Although fully active, *Pichia*-produced TmelEST2 migrated anomalously on SDS-polyacrylamide gels compared to the enzyme purified from *E. coli*. This anomalous electrophoretic behaviour was fully reverted by endo-β-N-acetylglucosaminidase H (Endo H, New England Biolabs) treatment (conducted according to the manufacturer's instructions), thus indicating co-secretory glycosylation of the enzyme produced in *P. pastoris*.

Supplementary References

1. Krissinel, E. & Henrick, K. Inference of macromolecular assemblies from crystalline state. *J Mol Biol* **372,** 774–797 (2007).
2. Dou, S. *et al.* Crystal structures of Pseudomonas putida esterase reveal the functional role of residues 187 and 287 in substrate binding and chiral recognition. *Biochem. Biophys. Res. Commun.* **446,** 1145–1150 (2014).
3. Yang, S., Qin, Z., Duan, X., Yan, Q. & Jiang, Z. Structural insights into the substrate specificity of two esterases from the thermophilic Rhizomucor miehei. *J Lipid Res* **56,** 1616–1624 (2015).
4. Palm, G. J. *et al.* The crystal structure of an esterase from the hyperthermophilic microorganism Pyrobaculum calidifontis VA1 explains its enantioselectivity. *Appl Microbiol Biotechnol* **91,** 1061–1072 (2011).
5. Ejima, K. *et al.* Molecular cloning and characterization of a thermostable carboxylesterase from an archaeon, Sulfolobus shibatae DSM5389: non-linear kinetic behavior of a hormone-sensitive lipase family enzyme. *J Biosci Bioeng* **98,** 445–451 (2004).
6. Byun, J. S. *et al.* Crystal structure of hyperthermophilic esterase EstE1 and the relationship between its dimerization and thermostability properties. *BMC Struct Biol* **7,** 47 (2007).
7. De Simone, G. *et al.* The crystal structure of a hyper-thermophilic carboxylesterase from the archaeon Archaeoglobus fulgidus. *J Mol Biol* **314,** 507–518 (2001).
8. De Simone, G. *et al.* A snapshot of a transition state analogue of a novel thermophilic esterase belonging to the subfamily of mammalian hormone-sensitive lipase. *J. Mol. Biol.* **303,** 761–771 (2000).
9. Schubot, F. D. *et al.* Structural Basis for the Substrate Specificity of the Feruloyl Esterase Domain of the Cellulosomal Xylanase Z from *Clostridium thermocellum* [†]. *Biochemistry* **40,**

12524–12532 (2001).

10. Tisserant, E. *et al.* Deep RNA sequencing improved the structural annotation of the Tuber melanosporum transcriptome. *New Phytol.* **189,** 883–891 (2011).

11. Martin, F. *et al.* Périgord black truffle genome uncovers evolutionary origins and mechanisms of symbiosis. *Nature* **464,** 1033–8 (2010).

12. Spagnoli, G. *et al.* Secretory production of designed multipeptides displayed on a thermostable bacterial thioredoxin scaffold in Pichia pastoris. *Protein Expression and Purification* (2016). doi:10.1016/j.pep.2016.04.012
